# Glucosamine stimulates pheromone-independent dimorphic transition in *Cryptococcus neoformans* by promoting Crz1 nuclear translocation

**DOI:** 10.1371/journal.pgen.1006982

**Published:** 2017-09-12

**Authors:** Xinping Xu, Jianfeng Lin, Youbao Zhao, Elyssa Kirkman, Yee-Seul So, Yong-Sun Bahn, Xiaorong Lin

**Affiliations:** 1 Center for Experimental Medicine, The First Affiliated Hospital of Nanchang University, Nanchang, Jiangxi, China; 2 Department of Biology, Texas A&M University, College Station, Texas, United States of America; 3 Department of Microbiology, University of Georgia, Athens, Georgia, United States of America; 4 Department of Biotechnology, Yonsei University, Seoul, Korea; Carnegie Mellon University, UNITED STATES

## Abstract

Morphotype switch is a cellular response to external and internal cues. The *Cryptococcus neoformans* species complex can undergo morphological transitions between the yeast and the hypha form, and such morphological changes profoundly affect cryptococcal interaction with various hosts. Filamentation in *Cryptococcus* was historically considered a mating response towards pheromone. Recent studies indicate the existence of pheromone-independent signaling pathways but their identity or the effectors remain unknown. Here, we demonstrated that glucosamine stimulated the *C*. *neoformans* species complex to undergo self-filamentation. Glucosamine-stimulated filamentation was independent of the key components of the pheromone pathway, which is distinct from pheromone-elicited filamentation. Glucosamine stimulated self-filamentation in H99, a highly virulent serotype A clinical isolate and a widely used reference strain. Through a genetic screen of the deletion sets made in the H99 background, we found that Crz1, a transcription factor downstream of calcineurin, was essential for glucosamine-stimulated filamentation despite its dispensability for pheromone-mediated filamentation. Glucosamine promoted Crz1 translocation from the cytoplasm to the nucleus. Interestingly, multiple components of the high osmolality glycerol response (HOG) pathway, consisting of the phosphorelay system and some of the Hog1 MAPK module, acted as repressors of glucosamine-elicited filamentation through their calcineurin-opposing effect on Crz1’s nuclear translocation. Surprisingly, glucosamine-stimulated filamentation did not require Hog1 itself and was distinct from the conventional general stress response. The results demonstrate that *Cryptococcus* can resort to multiple genetic pathways for morphological transition in response to different stimuli. Given that the filamentous form attenuates cryptococcal virulence and is immune-stimulatory in mammalian models, the findings suggest that morphogenesis is a fertile ground for future investigation into novel means to compromise cryptococcal pathogenesis.

## Author summary

Cryptococcal meningitis claims half a million lives each year. There is no clinically available vaccine and the current antifungal therapies have serious limitations. Thus identifying cryptococcal specific programs that can be targeted for antifungal or vaccine development is of great value. We have shown previously that switching from the yeast to the hypha form drastically attenuates/abolishes cryptococcal virulence. Cryptococcal cells in the filamentous form also trigger host immune responses that can protect the host from a subsequent lethal challenge. However, self-filamentation is rarely observed in serotype A isolates that are responsible for the vast majority of cryptococcosis cases. In this study, we found that glucosamine stimulated self-filamentation in genetically distinct strains of the *Cryptococcus* species complex, including the most commonly used serotype A reference strain H99. We demonstrated that filamentation elicited by glucosamine did not depend on the pheromone pathway, but it requires the calcineurin transcription factor Crz1. Glucosamine promotes nuclear translocation of Crz1, which is positively controlled by the phosphatase calcineurin and is suppressed by the HOG pathway. These findings raise the possibility of manipulating genetic pathways controlling fungal morphogenesis against diseases caused by the *Cryptococcus* species complex.

## Introduction

The opportunistic environmental fungal pathogen, *Cryptococcus neoformans*, is a leading killer of HIV-infected patients worldwide [1, 2]. It causes 1 million infections and more than half a million deaths each year worldwide [1]. *C*. *neoformans* is a species complex containing several serotypes (A, D, and AD hybrids) [3]. Among these serotypes, serotype A (*C*. *neoformans* var. *grubii*) causes approximately 95% of all cryptococcosis cases [4, 5], whereas serotype D (*C*. *neoformans* var. *neoformans*) is responsible for about 5% of the cases. The current anti-cryptococcal treatments rely primarily on azole antifungals with or without the induction therapy with amphotericin B [6]. The mortality rates of cryptococcosis are unacceptably high (~10–75%) [1, 7–9]. To further compound the problem, the emergence of resistance to azole drugs has been observed in multiple regions around the world [10–14] and relapse frequently occurs following treatment largely due to failure to clear the original infection [12, 15]. Thus, it is of great value to identify cryptococcal specific programs that can be used for new antifungal or vaccine development.

Morphotype switch between yeast and hypha is a cellular adaptation tightly linked to the virulence of dimorphic fungal pathogens [16–18]. In *Candida albicans*, proteins associated with hyphal growth are shown to impact its pathogenicity and some of them provide the bases for vaccine development [19–23]. Our previous studies in *C*. *neoformans* demonstrated that morphotypes (yeast or filament) are tightly linked to pathogenicity of this fungus as well [24–26]. Znf2, the master regulator of filamentation, is a potent anti-virulent factor. Deletion of this zinc finger transcription factor locks cells in yeast form and enhances fungal virulence in a murine model of cryptococcosis. Conversely, overexpression of *ZNF2* drives cells to the hyphal form and attenuates/abolishes the ability of *C*. *neoformans* to cause fatal infections [24–26]. *Cryptococcus* cells with *ZNF2* overexpression stimulate protective immune responses in the host and provide protection to the animal against a subsequent challenge by the highly virulent serotype A clinical isolate H99 [26]. These findings indicate that activation of the filamentation program could drastically compromise cryptococcal virulence. Thus, the yeast-to-hypha morphological transition provides an important avenue to explore alternative measures for the prevention and/or treatment of cryptococcal infections.

*C*. *neoformans* species is not considered a conventional dimorphic fungus due to the historical association of the yeast-to-hypha transition with mating. The mating response is controlled by the pheromone pathway composed of the pheromone, the pheromone receptor, the Cpk1 mitogen-activated protein kinase (MAPK) module, and the ultimate HMG domain transcription factor Mat2 [25, 27–32]. The pheromone pathway promotes self-filamentation during unisexual development or dikaryotic filamentation during **a**-α bisexual development. As expected, the pheromone pathway is activated under mating-inducing conditions (e.g. dehydration, nutrition limitation, V8 juice, and darkness). However, the host environment is not favorable for mating and the pheromone pathway exerts no or minimal impact on virulence [25, 29, 33, 34]. Recent studies with *C*. *neoformans* serotype D isolates indicate that the pheromone pathway is essential for filamentation during **a**-α bisexual mating [24, 25, 35–37], but it is not necessary for self-filamentation in a unisexual population under certain conditions [38–41]. Given the largely unisexual population of *C*. *neoformans* (α >99%, **a** <1%), it is important to identify pheromone-independent pathways that can control self-filamentation.

Although all serotypes of the *C*. *neoformans* species complex are expected to possess the ability to undergo self-filamentation, self-filamentation is often observed in serotype D isolates and rarely in serotype A isolates [42–45], including the highly virulent clinical isolate and the most widely used serotype A reference strain H99 [46–48]. This hinders the investigation of morphological transition in *C*. *neoformans* as many resources are generated for the H99 background, including a congenic pair, gene deletion sets, a well-annotated genome, and vast literatures about cryptococcal biology and pathology [49–52]. The rarity of self-filamentation in serotype A isolates challenge the possibility of mitigating the diseases caused by the *C*. *neoformans* species complex through activating the filamentation program.

Here, we found that glucosamine stimulated self-filamentation in both serotype D and serotype A strains, including H99. Although we found that both N-acetyl-glucosamine (GlcNAc) and glucosamine could stimulate filamentation in another fungal pathogen *C*. *albicans*, GlcNAc showed no effect on filamentation in *C*. *neoformans*. We demonstrated that filamentation in *C*. *neoformans* evoked by glucosamine was independent of the pheromone pathway. By genetic screens, we discovered that Crz1, a transcription factor downstream of calcineurin, was required for this process. The requirement of Crz1 for filamentation is specific to the response elicited by glucosamine, as Crz1 is not critical for filamentation elicited by pheromone [38]. We demonstrated that glucosamine promoted the translocation of Crz1 from the cytoplasm to the nucleus where it could exert its function as a transcription factor. Not surprisingly, we found that the catalytic and regulatory subunits of the phosphatase calcineurin, Cna1 and Cnb1, were essential for the nuclear translocation of Crz1 and for filamentation. Interestingly, multiple components in the HOG pathway, except Hog1 itself, acted as repressors of glucosamine-elicited filamentation through their calcineurin-opposing effect on Crz1’s nuclear translocation. Deletion of these kinases increased the basal level of nucleus-localized Crz1. These findings indicate that *C*. *neoformans* can resort to different genetic pathways for morphological transition in response to different stimuli, paving the way for future investigation to identify signals and targets that can be used to manipulate morphogenesis of this fungal pathogen *in vivo*.

## Results

### Glucosamine stimulates H99 and other strains to undergo self-filamentation

**Fig 1:**
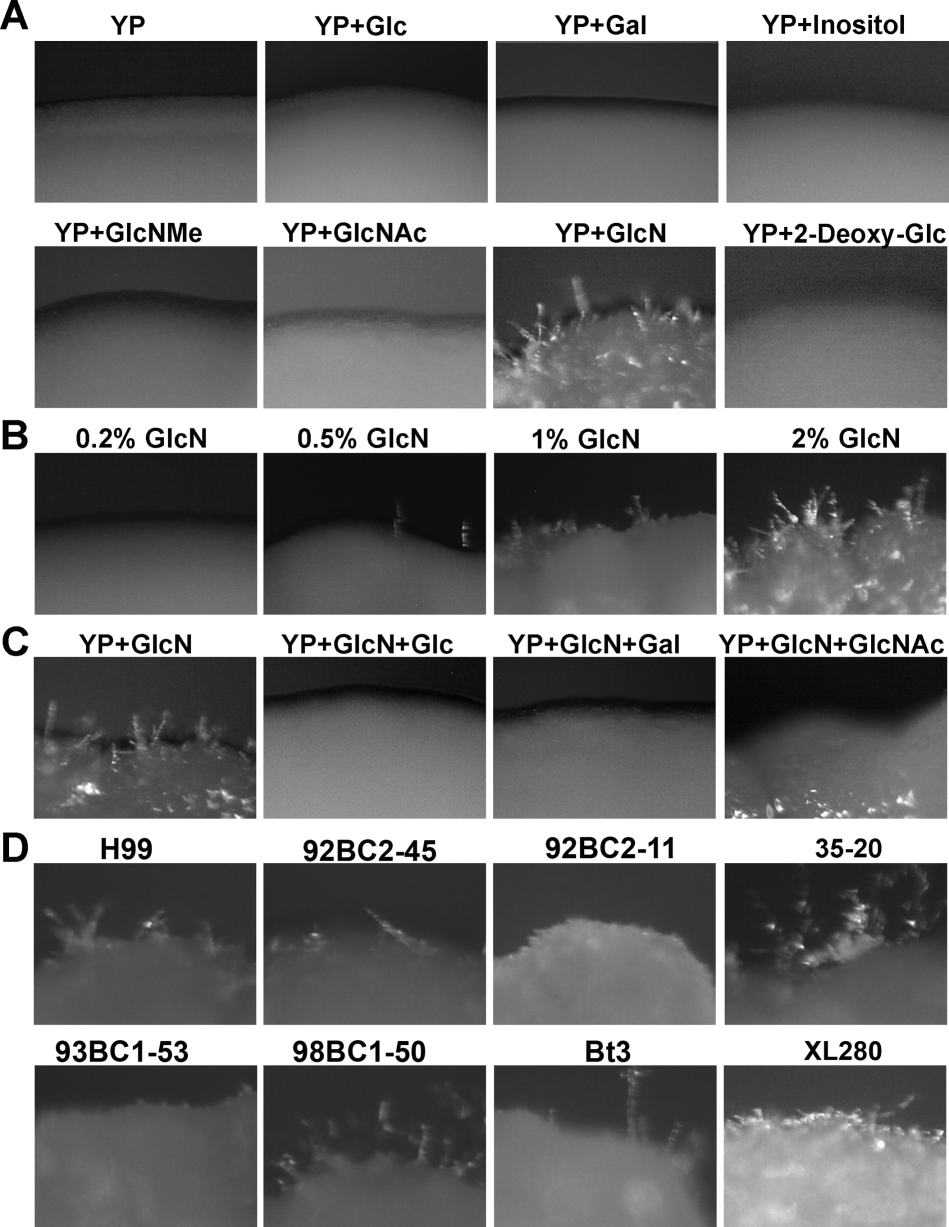
Glucosamine stimulates self-filamentation in H99 and other cryptococcal isolates. (**A**) The effect of the addition of different six-carbon sugars and hexamines at 2% to YP base medium. H99 was cultured on YP, YP+Glc (glucose), YP+Gal (galactose), YP+Inositol, YP+GlcNMe (N-Methyl-glucosamine), YP+GlcNAc (N-Acetyl-glucosamine), YP+GlcN (glucosamine), and YP+2-Dexoyl-Glc (2-Deoxyl-glucose) for 7 days. (**B**) The dose-dependent effect of glucosamine on self-filamentation in H99. H99 was cultured on YP+GlcN at final concentration of 0, 0.2%, 0.5%, 1%, and 2% for 7 days. (**C**) The inhibitory effect of other carbon sources on GlcN-induced filamentation. H99 was cultured on the indicated media for 7 days. H99 cultured on YP+GlcN (2%) was used as control. Other carbon sources, such as glucose, galactose, or N-Acetyl-glucosamine (2%) were added to the YP+GlcN medium. (**D**) The effect of glucosamine on filamentation is not specific to H99. 92BC2-45 (serotype A), 92BC2-11 (serotype A), 93BC1-53 (serotype D), 35–20 VNI (serotype A), 98BC1-50 (serotype D), Bt3 strain (serotype A), and XL280α (serotype D) were cultured on YP+GlcN medium for 7 days.

Wild-type H99 does not undergo self-filamentation under all mating-inducing conditions. Here, we decided to test the effect of different carbon sources based on previous studies in other dimorphic fungal pathogens such as *Candida albicans* [53–56], *Histoplasma capsulatum*, and *Blastomyces dermatitidis* [57] where N-acetyl-glucosamine (GlcNAc) activates hyphal growth. Here, we used YP medium (1% yeast extract and 2% peptone) as the base medium and supplemented it with different carbon sources at the final concentrations of 2%. We included 6-carbon sugars (glucose, galactose, and inositol), amino sugars (N-methyl-glucosamine, N-acetyl glucosamine, and glucosamine), and other carbon sources (glycerol, ethanol, and sodium acetate). None of the carbon sources tested stimulated filamentation in H99, with the exception of glucosamine (Fig 1A). Filamentation induced by glucosamine was unlikely to be an effect of carbon repression, as the non-metabolizable glucose analog 2-deoxyl glucose did not trigger filamentation in H99 (Fig 1A). The filamentation stimulated by glucosamine was also unlikely to be a general effect due to the activation of the hexamine metabolism pathway, as N-methyl-glucosamine and N-acetyl glucosamine both failed to stimulate filamentation in H99 (Fig 1A).

The effect of glucosamine on filamentation was dose-dependent, as glucosamine at lower concentrations (<0.5%) did not evoke obvious hyphal growth in H99 (Fig 1B). Robust filamentation was observed when glucosamine was present at concentrations higher than 1% (Fig 1B). The addition of other carbon sources (e.g. glucose, galactose, or GlcNAc) inhibited filamentation in H99 (Fig 1C). This suggests potential competitive inhibition of glucosamine by other carbon sources. Although not all strains could produce hyphae on the glucosamine medium, glucosamine-stimulated filamentation was not limited to H99. Some other isolates of either serotype A or serotype D (e.g. XL280) self-filamented on glucosamine medium (Fig 1D). Interestingly, glucosamine-stimulated filamentation not only in *C*. *neoformans*, but also in some *C*. *albicans* strains (S1 Fig). This suggests that glucosamine could be a general signal for fungal morphogenesis.

### Glucosamine-stimulated filamentation requires Znf2 but not the pheromone sensing pathway controlled by Mat2

*C*. *neoformans* typically undergoes yeast-to-hypha transition during **a**-α bisexual mating or during unisexual development. Two transcription factors, Mat2 and Znf2, were demonstrated to be critical for hyphal growth during sexual development [24, 25, 37]. Mat2 controls the pheromone pathway and plays a central role in cell fusion [25]. Under mating-inducing conditions (e.g. on V8 medium), Mat2 activates Znf2, the master regulator of filamentation [24, 25]. However, under mating-suppressing conditions (e.g. on YPD medium), overexpression of *MAT2* fails to activate Znf2 despite high levels of pheromone and *C*. *neoformans* remains in the yeast form [24].

**Fig 2:**
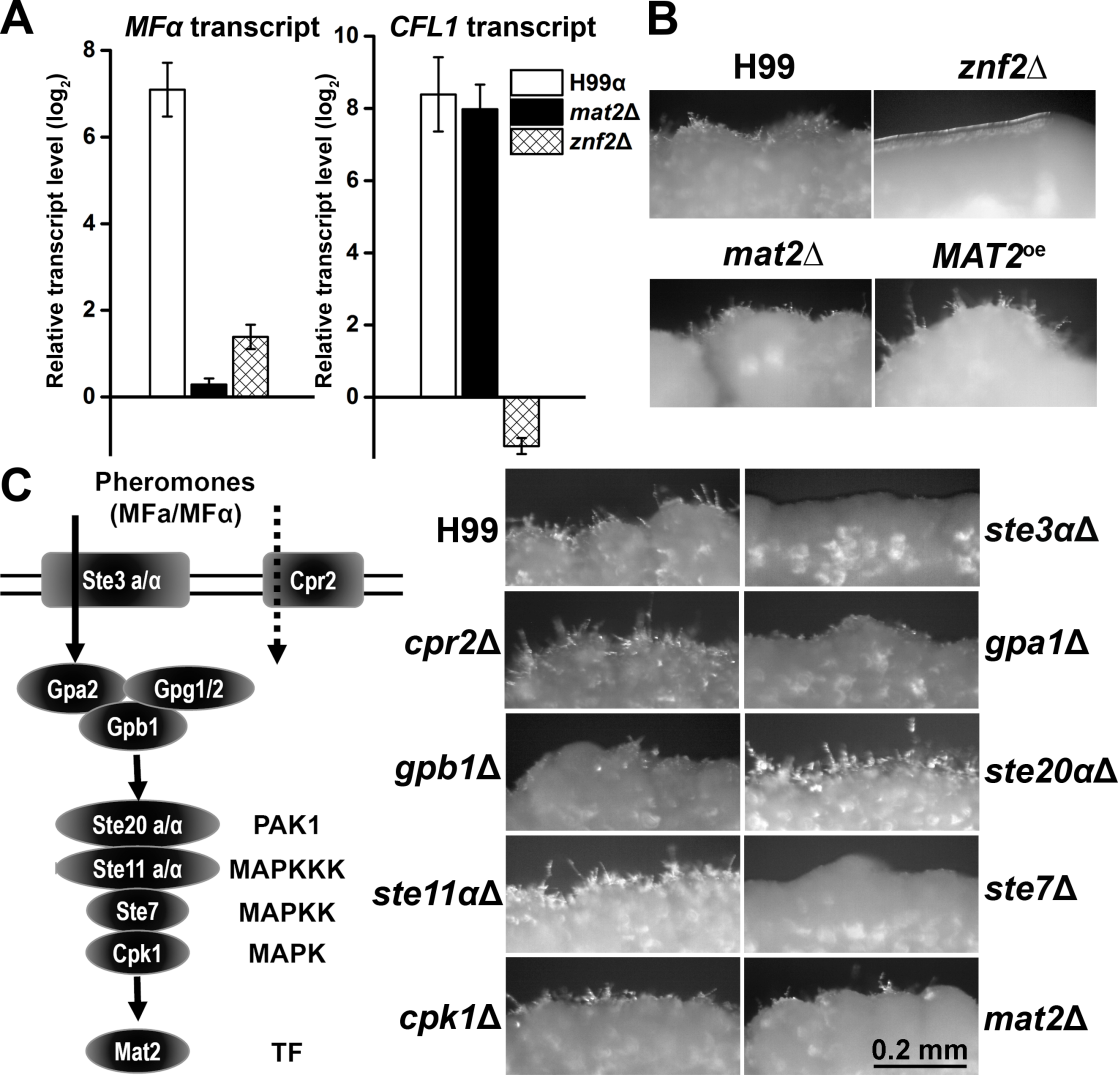
Glucosamine-stimulated filamentation is independent of the Mat2-controlled pheromone pathway but requires the morphogenesis regulator Znf2. (**A**) The *CFL1* and *MFα* transcript levels in wild-type H99, the *znf2*Δ mutant, and the *mat2*Δ mutant on glucosamine medium for 4 days relative to those on the control YP medium. The house keeping gene *TEF1* is used as the internal control. (**B**) The wild-type H99 strain, the *znf2*Δ mutant, the *mat2*Δ mutant, and the *MAT2*^*oe*^ strain were cultured on YP+GlcN medium for 6 days. (**C**) The left panel depicts some of the key components of the pheromone pathway. The right panel shows images of colonies of wild-type H99, the *ste3*Δ mutant, the *cpr2*Δ mutant, the *gpa1*Δ mutant, the *gpb1*Δ mutant, the *ste20*αΔ mutant, the *ste11*αΔ mutant, the *ste7*Δ mutant, the *cpk1*Δ mutant, and the *mat2*Δ mutant cultured on medium for 7 days.

We first examined the effect of glucosamine on the activity of Mat2 and Znf2 in wild-type H99. We measured the transcript levels of their target genes, which reflected the activities of these transcription factors. The pheromone gene *MFα* is the most upregulated gene controlled by Mat2 during both bisexual and unisexual development [25, 37]. The filamentation marker gene *CFL1* is one of the highly expressed genes upregulated by Znf2 [24, 58, 59]. We found that the transcript levels of *MFα* and *CFL1* increased more than 100 and 300 folds respectively when H99 was cultured on glucosamine medium compared to that of the base medium at 96 hours (Fig 2A), indicating the activation of both Mat2 and Znf2.

To examine if self-filamentation in H99 evoked by glucosamine relies on Mat2 or/and Znf2, we tested the *znf2*Δ mutant and the *mat2*Δ mutant on glucosamine medium. As expected, the *MFα* transcript level was no longer induced by glucosamine in the *mat2*Δ mutant (Fig 2A). By contrast, a strong induction of the filamentation marker *CFL1* at a level comparable to that in wild-type H99 was observed in the *mat2*Δ mutant (Fig 2A). Consistent with the expression of the filamentation marker *CFL1*, the *mat2*Δ mutant self-filamented on glucosamine medium (Fig 2B). A strain overexpressing *MAT2* (*MAT2*^*oe*^) also self-filamented on glucosamine medium. The result indicates that Mat2 is not essential for glucosamine-stimulated filamentation.

In contrast to the *mat2*Δ mutant, there was no increase but rather a modest reduction in the *CFL1* transcript level in the *znf2*Δ mutant on glucosamine medium compared to that of the base medium (Fig 2A). The *MFα* transcript level increased slightly (~4 fold) in this mutant (Fig 2A). The low level of *CFL1* in the *znf2*Δ mutant was consistent with its non-filamentous phenotype on glucosamine medium (Fig 2B). Collectively, these observations indicate that Znf2, but not Mat2, is required for glucosamine-stimulated filamentation in H99. The self-filamentation observed in H99 and the *mat2*Δ mutant was a response to glucosamine. The wild-type H99, the *mat2*Δ mutant, or the *znf2*Δ mutant is incapable of self-filamentation on V8 medium (S2 Fig).

To determine if the dispensability of Mat2 and the essentiality of Znf2 in glucosamine-stimulated filamentation are conserved in *C*. *neoformans*, we further tested the *mat2*Δ mutant and the *znf2*Δ mutant made in the serotype D XL280 background. No hyphal growth was observed in the *znf2*Δ mutant while the *mat2*Δ mutant filamented similarly as the wild-type control on glucosamine medium (S2 Fig). This is again different from filamentation observed on mating-inducing V8 medium where Mat2 is required (S2 Fig)[25, 38]. This result corroborates the conclusion that filamentation elicited by glucosamine requires the morphogenesis regulator Znf2, but not the pheromone pathway regulator Mat2.

To further verify that the pheromone pathway is not critical for glucosamine-stimulated filamentation, we tested additional mutants in the H99 background with disruption in the following key components of this pathway (Fig 2C), namely the pheromone receptor Ste3 [60, 61], the pheromone receptor like protein Cpr2 [62], Gβ subunit Gpb1 [64], a PAK kinase Ste20α [65], the MAPK kinase kinase Ste11α [33], the MAPK kinase Ste7 [32], and the MAPK Cpk1 [32]. We also included Gα subunit Gpa1 [63] that regulates mating through the cAMP/PKA pathway in our test. Except for the *ste3α*Δ and the *ste7*Δ mutants, all other gene deletion mutants tested filamented on glucosamine medium (Fig 2C). The *ste3α*Δ could eventually produce some filaments after prolonged incubation. To verify that the blocked filamentation observed in the *ste7*Δ mutant is not an artifact, we tested multiple *ste7*Δ isolates generated in both mating type **a** and α backgrounds. All the *ste7*Δ mutants tested showed only yeast growth on glucosamine medium, indicating the unique role of Ste7 in filamentation compared to other components of the pheromone pathway. Collectively, the results indicate that the pheromone pathway overall is dispensable for filamentation induced by glucosamine.

### Identify the possible pathways involved in glucosamine-stimulated self-filamentation through genetic screens

To identify genes that are involved in filamentation triggered by glucosamine, we screened approximately 2500 gene deletion mutants made in the H99 background for altered filamentation on glucosamine medium. The strains screened included the partial genome deletion set generated by Dr. Hiten Madhani’s group in 2015, and the transcription factor and the kinase deletion sets generated by Dr. Yong-Sun Bahn’s group [66, 67]. Among the deletion mutants tested, two genes encoding the glucosamine-6-phosphate deaminase Gnd1 (gene locus # CNAG_06098) and the glucosamine 6-phosphate N-acetyltransferase Gnat1 (gene locus # CNAG_05695) are involved in the hexamine metabolism pathway (S3A Fig). The *gnd1*Δ mutant was unable to grow in the presence of glucosamine (even at concentrations lower than 0.1%) (S3B Fig), suggesting that the Gnd1 is essential for the growth of *C*. *neoformans* under such conditions. *GNAT1* was not essential for growth on glucosamine medium. However, the *gnat1*Δ mutant filamented as well as, if not better than, the wild-type H99 on glucosamine medium (S3C Fig). The result suggests that the hexamine metabolism is unlikely to be responsible for the filamentous growth elicited by glucosamine.

**Fig 3:**
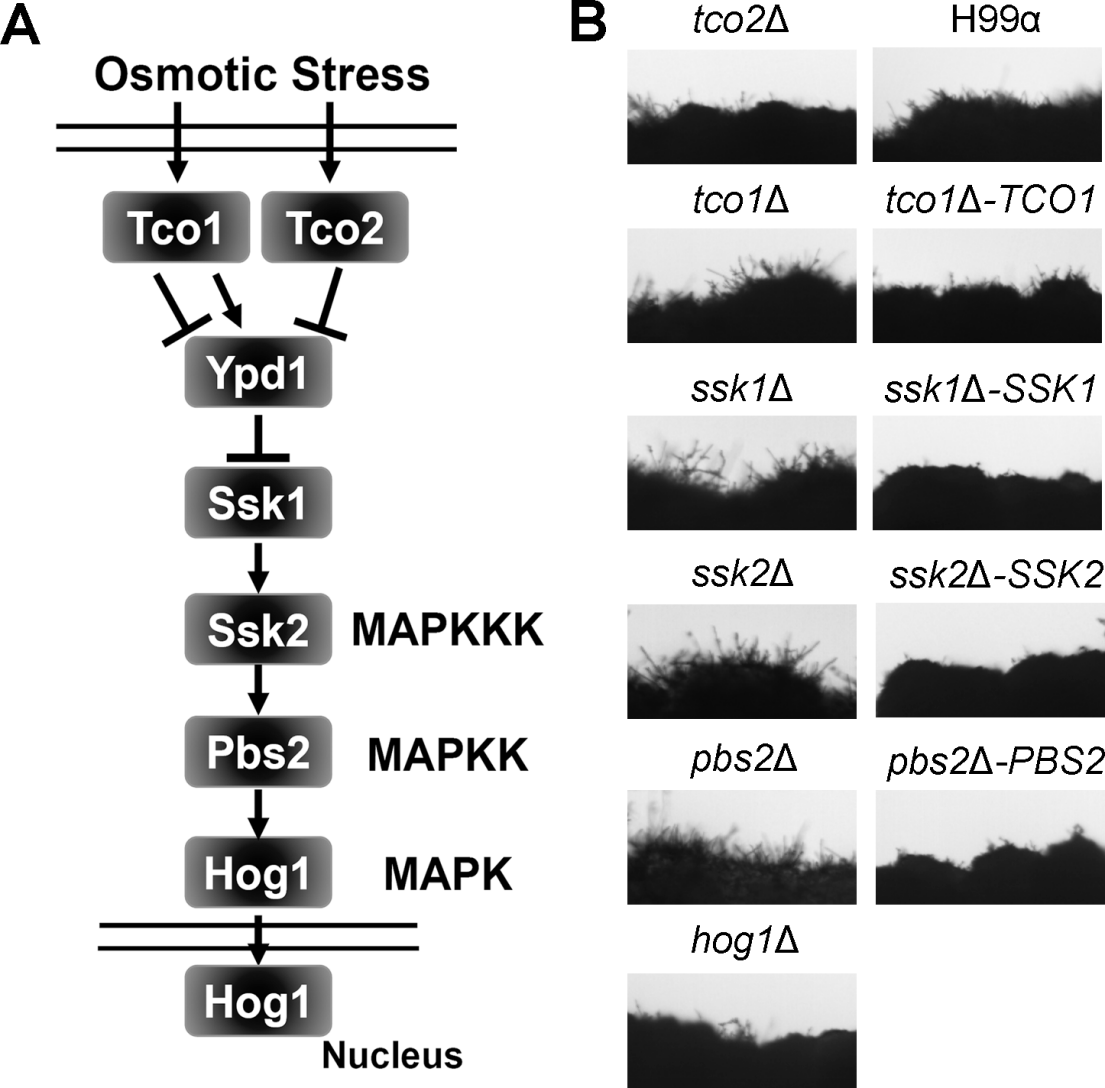
Multiple components of the HOG pathway suppress filamentation on glucosamine medium. (**A**) A diagram depicting the major components of the osmotic sensing HOG pathway in *Cryptococcus*. (**B**) The wild-type H99, the *tco1*Δ mutant, the *tco1*Δ-*TCO1* complemented strain, the *tco2*Δ mutant, the *ssk1*Δ mutant, the *ssk1*Δ-*SSK1* complemented strain, the *ssk2*Δ mutant, the *ssk2*Δ-*SSK2* complemented strain, the *pbs2*Δ mutant, the *pbs2*Δ-*PBS2* complemented strain, and the *hog1*Δ mutant were cultured on glucosamine medium (1.6% glucosamine) for 7 days.

We classified the mutants screened with altered filamentation into four groups: non-filamentous group, decreased filamentation, increased filamentation, and hyper-filamentation (S4 Fig and S1 Table). The fact that mutants with reduced/abolished filamentation and mutants with enhanced filamentation were recovered from the screen indicates that there are both repressors and activators of filamentation in response to glucosamine. Among mutants with enhanced filamentation, several were in the HOG pathway [68, 69] (Fig 3A), including the *tco1*Δ, *ssk1*Δ, *ssk2*Δ, and *pbs2*Δ mutants (Fig 3B). However, disruption of Hog1 itself, the downstream MAPK of this pathway, did not impact filamentation (Fig 3B). This observation suggests that glucosamine may not trigger the same response as osmotic stress. Consistent with this idea, the *crz1*Δ mutant is as resistant to osmotic stress caused by NaCl as the wild type (more details later. See S8 Fig).

**Fig 4:**
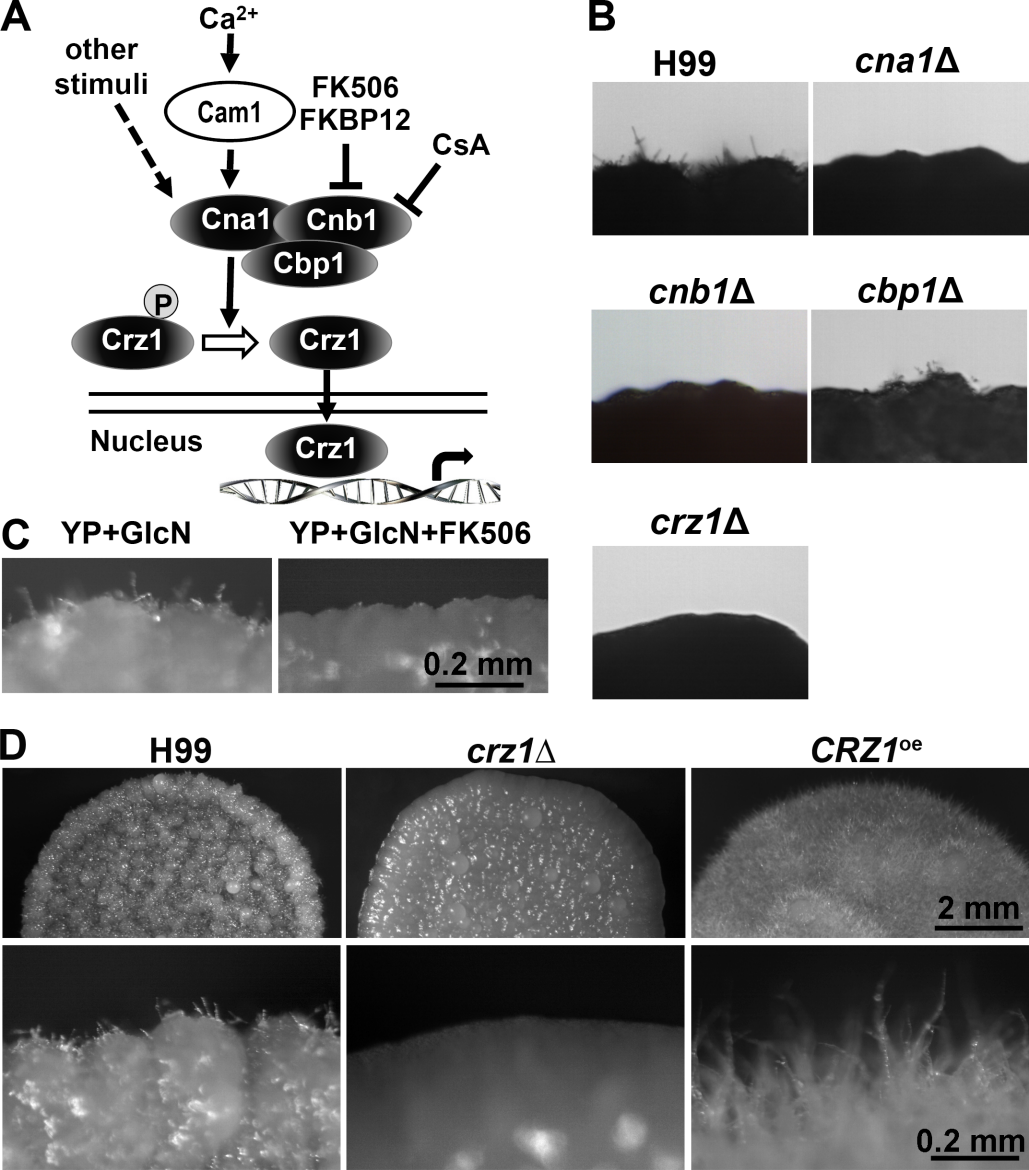
The calcineurin pathway is required for glucosamine-induced self-filamentation. (**A**) A diagram depicting the calcineurin pathway. (**B**) The wild-type H99, the *cna1*Δ mutant, the *cnb1*Δ mutant, the *cbp1*Δ mutant, and two independent *crz1*Δ mutants were cultured on glucosamine medium for 7 days. (**C**) The wild-type H99 was cultured on glucosamine medium with or without the calcineurin inhibitor FK506. (**D**) The wild-type H99, the *crz1*Δ mutant, and the *CRZ1*^*oe*^ strain (P_*GPD1*_-*CRZ1*) were cultured on glucosamine medium for 7 days.

Among the gene deletion mutants that showed blocked filamentation on glucosamine medium were the calcineurin mutants. These include the mutants with disruption in genes encoding the calcineurin catalytic subunit Cna1 [70, 71], the calcineurin regulatory subunit Cnb1 [70, 72], and the calcineurin downstream zinc finger transcription factor Crz1 (aka Sp1) [73–75] (Fig 4A and 4B, S1 Table). The mutant defective in the calcineurin binding protein Cbp1 [76, 77] showed reduced filamentation (Fig 4B). Treatment with the calcineurin inhibitor FK506 blocked the wild-type H99 from undergoing filamentation on glucosamine medium (Fig 4C), a phenotype similar to the *cna1*Δ, *cnb1*Δ, and *crz11*Δ mutants (Fig 4B).

Thus, the two pathways appear to exert opposing effects on glucosamine-stimulated self-filamentation in H99: the phosphorelay system and Ssk2-Pbs2 upstream of the Hog1 MAPK pathway suppress filamentation while the calcineurin pathway is required for filamentation.

### Crz1 controls glucosamine-stimulated filamentation and functions upstream of Znf2

Calcineurin transduces signals (e.g. elevated level of calcium) by dephosphorylating the downstream targets (Fig 4A). The transcription factor Crz1 is one of the targets of calcineurin, and not all responses controlled by calcineurin depend on Crz1 [78, 79]. For instance, the *cna1*Δ and *cnb1*Δ mutants displayed severe growth defect at 37°C and these mutants were hyper-sensitive to cell wall stress induced by Calcofluor White or Congo red [73–75, 78] (S5 Fig). By contrast, the *crz1*Δ mutant showed only slightly increased sensitivity to cell wall stress and heat stress (S5 Fig). Nonetheless, the *crz1*Δ mutant, like the calcineurin mutants (*cna1*Δ and *cnb1*Δ), was abolished in filamentation induced by glucosamine (Fig 4B). This suggests that Crz1 is a major effector of the calcineurin pathway in regulating filamentation in response to glucosamine.

**Fig 5:**
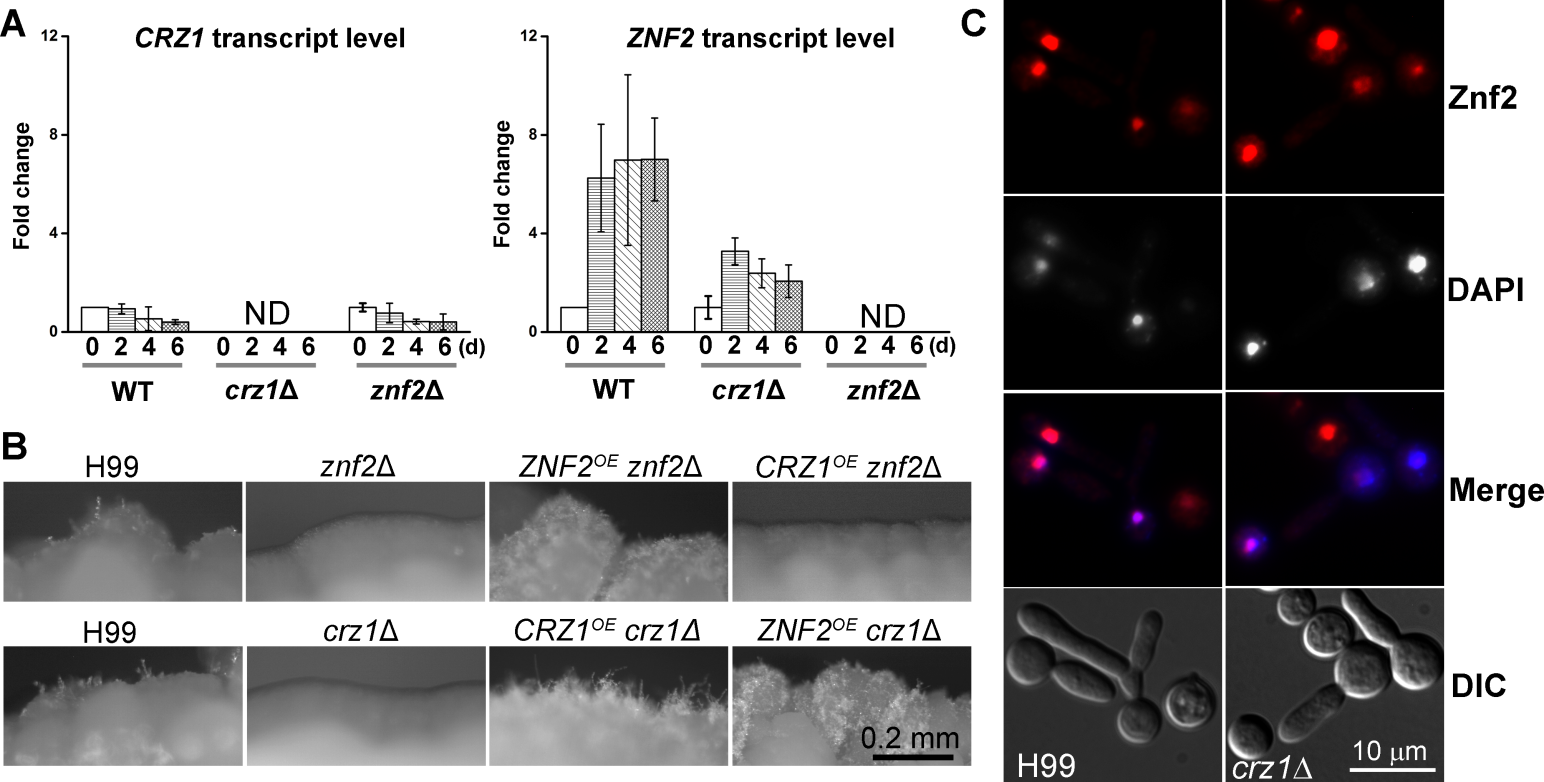
Crz1 acts upstream of Znf2 in regulating filamentation induced by glucosamine. (**A**) Transcript levels of *CRZ1* and *ZNF2* in the wild-type H99, the *crz1*Δ mutant, and the *znf2*Δ mutant cultured on glucosamine medium for 2 days, 4 days, and 6 days compared to the control of 0 days. (**B**) The wild-type H99, the *znf2*Δ mutant, the *ZNF2*^*oe*^ *znf2*Δ strain, the *CRZ1*^*oe*^ *znf2*Δ strain, the *crz1*Δ mutant, the *ZNF2*^*oe*^ *crz1*Δ strain, and the *CRZ1*^*oe*^ *crz1*Δ strain were cultured on glucosamine medium for 7 days. The overexpression of both *CRZ1* and *ZNF2* was driven by the constitutively active *GPD1* promoter and the inducible *CTR4* promoter respectively. (**C**) The subcellular localization of mCherry-Znf2 in the *crz1*Δ mutant and in the wild-type strain H99 on glucosamine medium. DAPI was used to indicate nuclear localization.

We then examined if overexpression of *CRZ1* could promote filamentation on glucosamine medium. To this end, we placed the *CRZ1* gene under the control of the constitutively active *GPD1* promoter [24, 80]. We introduced these constructs into the wild-type H99 or the *crz1*Δ mutant. We found that overexpression of *CRZ1* enhanced filamentation (Fig 4D and Fig 5B). The enhancement in filamentation by *CRZ1* overexpression was specific to the induction by glucosamine, as *CRZ1* overexpression did not confer self-filamentation to either wild-type H99 or the corresponding *crz1*Δ mutant when cells were cultured alone on V8 medium (S6A Fig). Furthermore, the deletion of *CRZ1* or the overexpression of *CRZ1* did not affect the ability of the strain to cross with a wild-type partner of the opposite mating type based on the observation that there was no notable difference between the crosses *crz1*Δ α x **a**, *CRZ1*^oe^ α x **a**, and α x **a** (S6B Fig). These findings suggest that alteration of the expression level of *CRZ1* does not impact mating efficiency controlled by the pheromone pathway, consistent with the recent finding in a serotype D strain [38].

To examine the genetic relationship between Crz1 and Znf2 in the regulation of filamentation in response to glucosamine, we first measured the transcript levels of *ZNF2* and *CRZ1* in the *znf2*Δ mutant and the *crz1*Δ mutant. The *CRZ1* transcript level on glucosamine medium was comparable to that of the base medium in wild type and its transcript level was also comparable between the wild type and the *znf2*Δ mutant (Fig 5A). This result indicates that neither Znf2 nor glucosamine has much impact on *CRZ1* at the transcript level. On the other hand, the *ZNF2* transcript level in wild-type H99 increased more than 6 fold on glucosamine medium and the degree of induction was much reduced in the *crz1*Δ mutant (2–3 fold) (Fig 5A). This suggests that deletion of *CRZ1* attenuated the induction of *ZNF2* elicited by glucosamine. Furthermore, overexpression of *CRZ1* failed to confer filamentation to the *znf2*Δ mutant while overexpression of *ZNF2* restored filamentation in the *crz1*Δ mutant on glucosamine medium (Fig 5B). Collectively, these epistatic results indicate that Crz1 functions upstream of Znf2 in response to glucosamine.

We then examined if disruption of Crz1 affects the subcellular localization of Znf2 after the Znf2 protein is made. For this purpose, we introduced the P_*CTR4*_-mCherry-*ZNF2* construct into the *crz1*Δ mutant and the wild-type H99 background. The mCherry-Znf2 signal was localized to the nucleus in both the *crz1*Δ mutant background and the wild-type background (Fig 5C). Collectively, the results suggest that Crz1 regulates *ZNF2* at the transcript level and it functions upstream of Znf2, and Crz1 does not affect the subcellular localization of the Znf2 protein.

### Crz1 translocates from the cytosol to the nucleus in response to glucosamine

**Fig 6:**
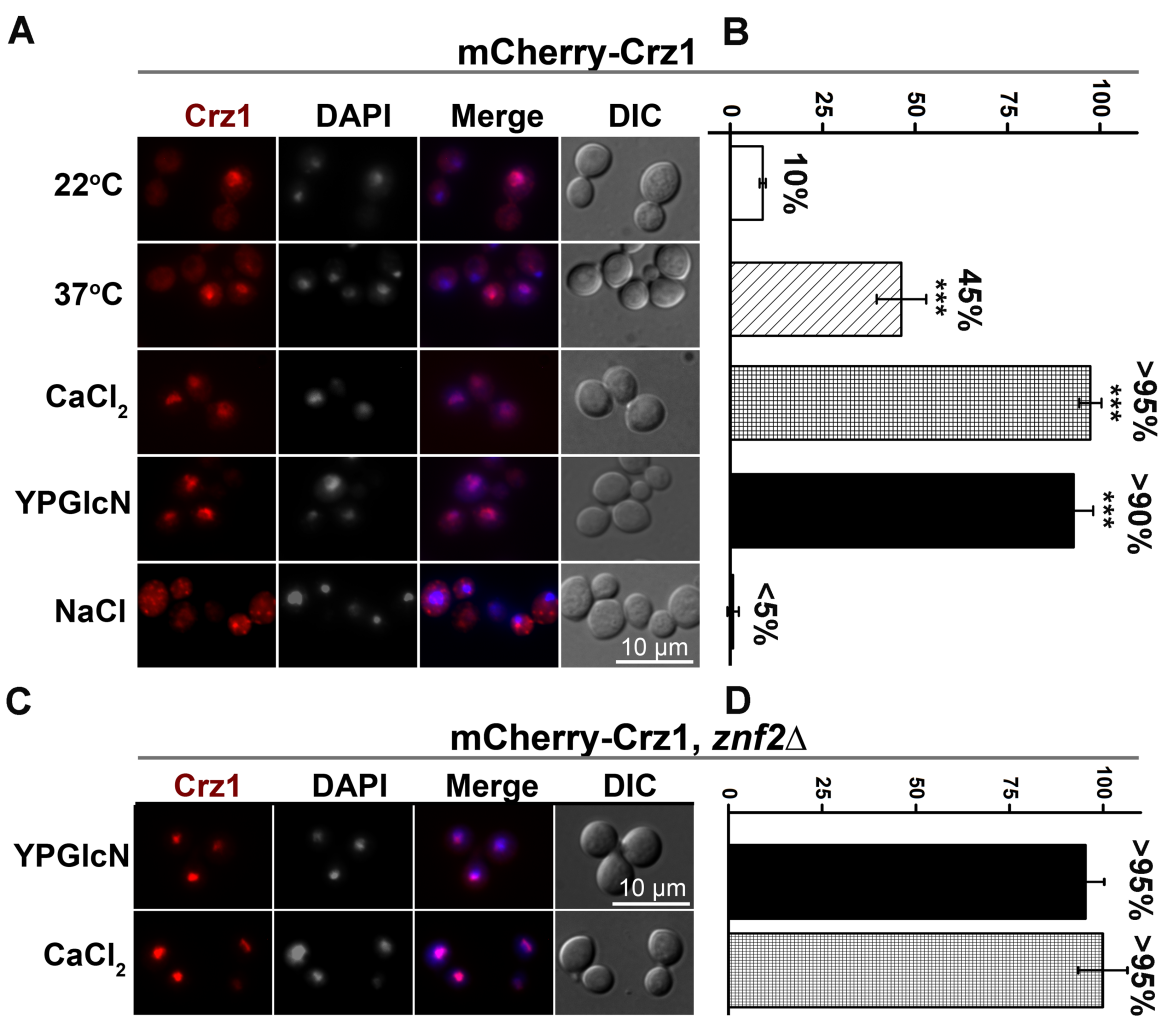
Glucosamine stimulates Crz1 translocation to the nucleus. (**A**) Localization of mCherry-Crz1 under different the indicated conditions. To test temperature’s effect on the subcellular localization of mCrz1, the strain P_*GPD1*_-mCherry-*CRZ1* was cultured in YPD liquid at 22°C (first row, or 37°C (2^nd^ row) with shaking overnight. To test the effect of calcium or NaCl on the subcellular localization of mCrz1, cells of the strain P_*GPD1*_-mCherry-*CRZ1* were collected from an overnight culture in liquid YPD at 22°C and then suspended in 100 mM CaCl_2_ or 1.5 M NaCl solution for 10–20 min (3^rd^ and 5^th^ rows). To test the effect of glucosamine on mCrz1’s localization, the strain P_*GPD1*_-mCherry-*CRZ1* was cultured in YPGlcN liquid medium for 12 hours at 22°C (4^th^ row). (**B**) Quantification of the percent of cells with mCherry-Crz1 located to the nucleus under the conditions used in panel A. (n≥60) (*** p<0.001). (**C**) Cells of the strain mCherry-*CRZ1*/*znf2*Δ were tested for the effect of glucosamine and CaCl_2_ on the localization of mCherry-Crz1 as described in panel A. (**D**) Quantification of the percentage of cells with mCherry-Crz1 located to the nucleus under the same conditions used in panel C.

Calcineurin is known to dephosphorylate Crz1 in response to certain stimuli like calcium or heat shock. Dephosphorylation causes the translocation of Crz1 from the cytosol to the nucleus for it to function as a transcription factor in *C*. *neoformans* [73–75] (Fig 4A). To examine if glucosamine affects the subcellular translocation of Crz1, we placed mCherry tagged Crz1 under the control of the constitutively active *GPD1* promoter. The exposure to either calcium or high temperature, two known stimuli of calcineurin, indeed stimulated mCherry-Crz1 in this overexpression strain to relocate from the cytosol into the nucleus (Fig 6A). As reported previously [75], NaCl induced granular localization of Crz1 in the cytosol (Fig 6A), indicating that the nuclear translocation of Crz1 is stimulus-specific. We then tested the effect of glucosamine on the subcellular localization of Crz1. Remarkably, greater than 90% of the cryptococcal population showed nuclear localization of Crz1 in the presence of glucosamine (Fig 6A and 6B). This indicates that glucosamine, like calcium, greatly increases the translocation of Crz1 from the cytosol into the nucleus. The translocation of Crz1 to the nucleus in response to calcium and glucosamine was not affected by Znf2 (Fig 6C and 6D), consistent with Crz1 functioning upstream of Znf2. To verify that the nuclear translocation effect of glucosamine was not an artifact due to *CRZ1* overexpression, we tested Crz1-mCherry placed under the control of its native promoter that was used in a recent study [79]. Again, glucosamine greatly increased the population of cryptococcal cells with nucleus-localized Crz1, as did calcium and the exposure to high temperature (S7 Fig). This finding indicates that glucosamine enhances nuclear translocation of the Crz1 protein regardless of its gene expression level.

**Fig 7:**
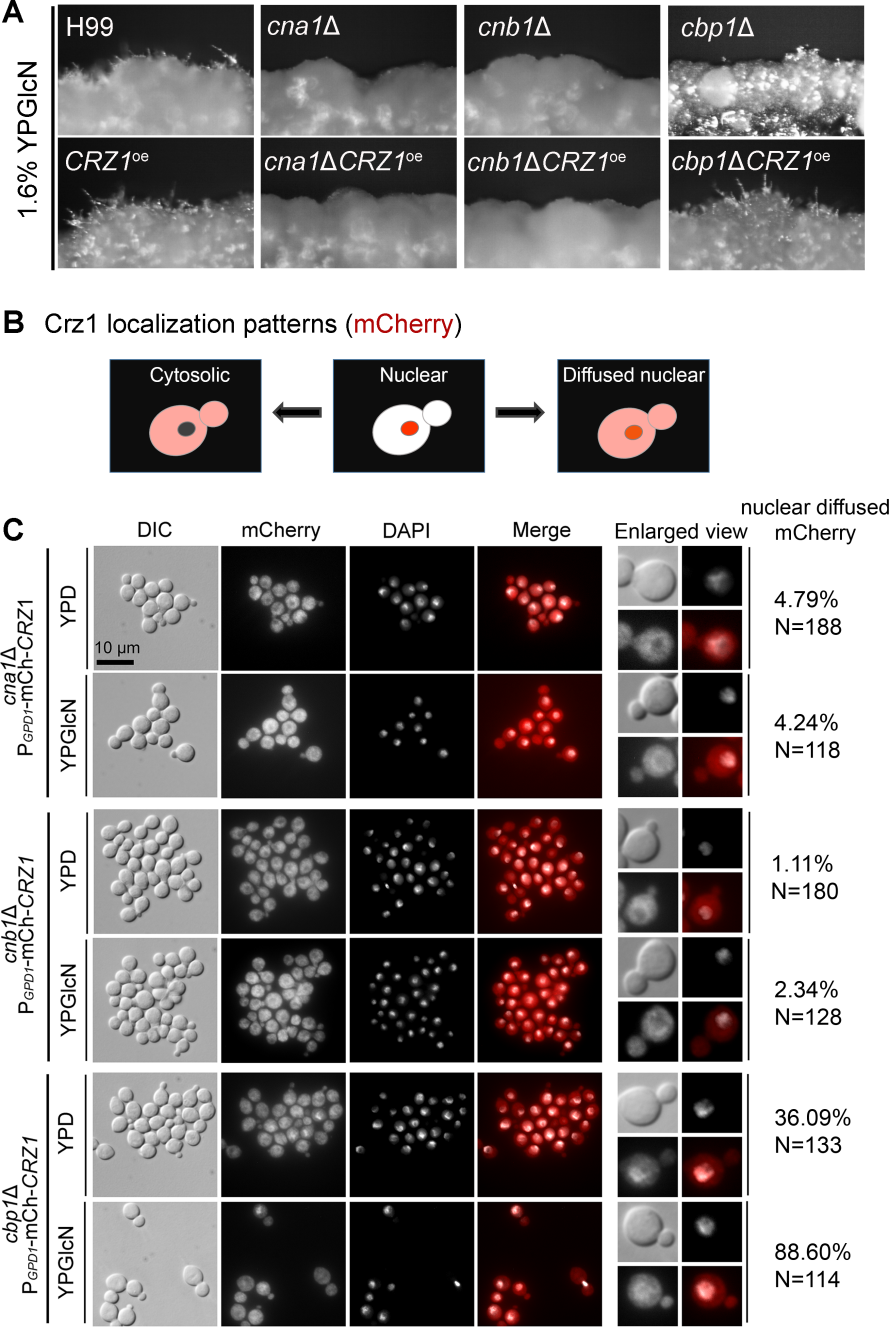
Crz1 depends on calcineurin for its nuclear localization and its regulation of filamentation on glucosamine medium. (**A**) Strains *cna1*Δ, *cna1*ΔP_*GPD1*_-mCherry-Crz1, *cnb1*Δ, *cnb1*ΔP_*GPD1*_-mCherry-Crz1, *cbp1*Δ, and *cbp1*ΔP_*GPD1*_-mCherry-Crz1 were cultured on YP+GlcN medium for 7 days. (**B**) a diagram depicting the different localization patterns of Crz1: diffused in the cytosol with nuclear exclusion (left); localized to the nucleus (middle); localized to both cytoplasm and the nucleus but more concentrated in the nucleus (right), (**C**) Strains *cna1*ΔP_*GPD1*_-mCherry-Crz1, *cnb1*ΔP_*GPD1*_-mCherry-Crz1, and *cbp1*ΔP_*GPD1*_-mCherry-Crz1 were cultured either in YPD medium or in YP+GlcN medium overnight. The mCherry-Crz1 signal showed diffused cytoplasmic localization in the *cna1*Δ and *cnb1*Δ mutants under both conditions. The mCherry-Crz1 signal showed diffused cytoplasmic localization but with more concentrated signals in the nucleus in the *cbp1*Δ mutant.

If glucosamine activates filamentation through its effect on the translocation of Crz1, we hypothesized that overexpression of *CRZ1* would be futile in the absence of a functional calcineurin. Indeed, no filamentation was observed when *CRZ1* was overexpressed in the *cna1*Δ mutant or the *cnb1*Δ mutant (Fig 7A). Similarly, overexpression of *CRZ1* did not restore the temperature sensitivity of the *cna1*Δ mutant (S8 Fig). Consistent with our hypothesis, Crz1 showed only cytoplasmic localization in the calcineurin *cna1*Δ and *cnb1*Δ mutants, regardless whether the cells were cultured in YPD medium or in glucosamine medium (Fig 7B and 7C). The *cbp1*Δ mutant showed reduced filamentation and overexpression of *CRZ1* in *cbp1*Δ restored filamentation (Fig 7A). Consistently, Crz1 was more concentrated in the nucleus in this mutant background (Fig 7B and 7C). The results demonstrate the essential role of calcineurin in controlling the nuclear translocation of Crz1, which correlates with robustness in filamentation.

### Components of the HOG pathway suppress filamentation by regulating the subcellular localization of Crz1

**Fig 8:**
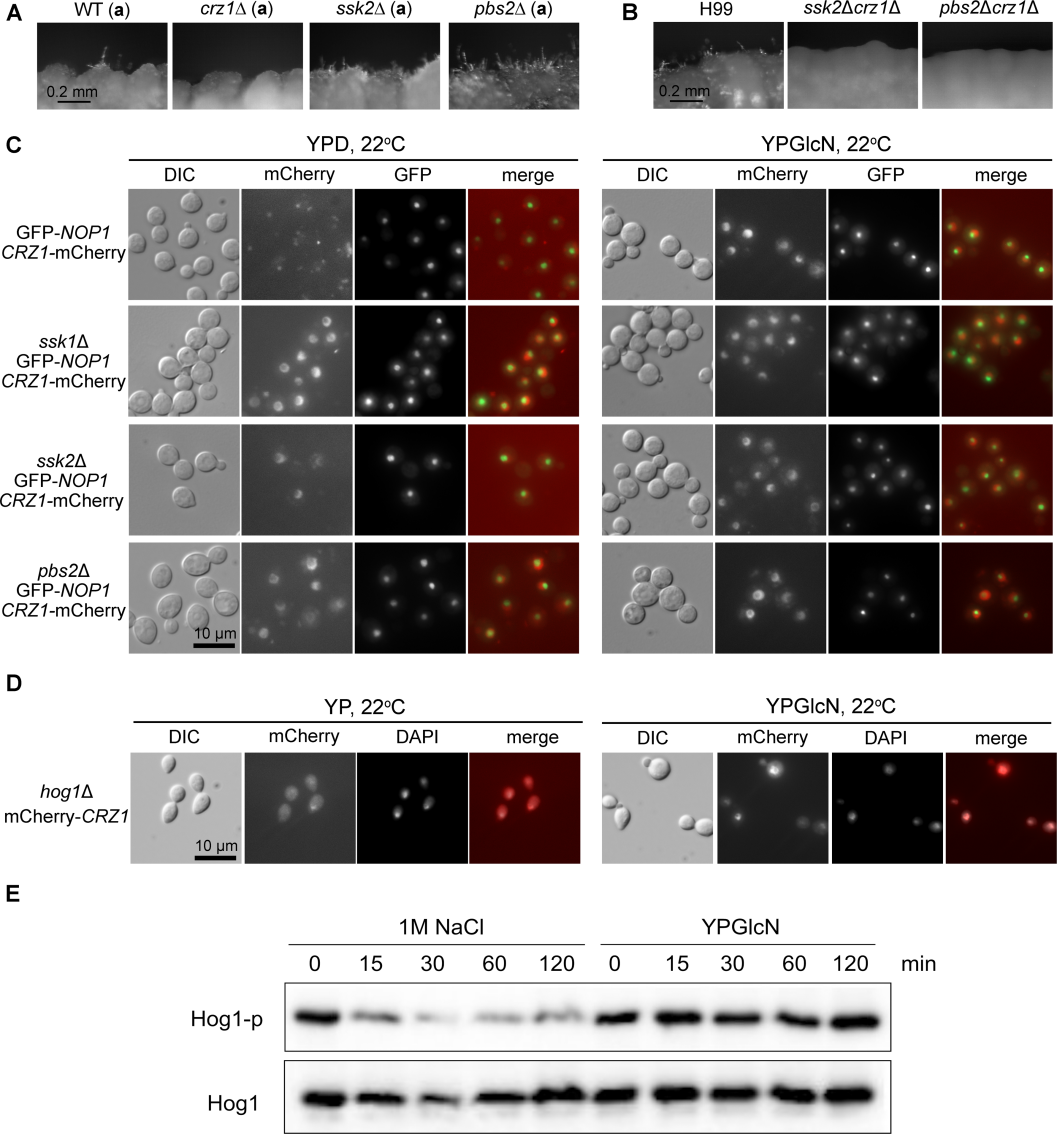
The HOG components function upstream of Crz1 and suppress Crz1’s nuclear localization. (**A**) Cells of the wild-type KN99**a**, *crz1*Δ (**a**), *ssk2*Δ (**a**), and *pbs2*Δ (**a**) were cultured on YP+GlcN (0.5%) medium for 7 days. (**B**) Cells of the wild-type H99, the *ssk2*Δ*crz1*Δ double mutant, and the *pbs2*Δ*crz1*Δ double mutant were cultured on YP+GlcN medium for 7 days. (**C**) The control strain XW252 (GFP-Nop1, P_*CRZ1*_-Crz1-mCherry) [79] and the corresponding *ssk1*Δ, *ssk2*Δ, and *pbs2*Δ mutants in the XW252 background were cultured in YPD or YP+GlcN medium overnight at 22°C. GFP-Nop1 labels the nucleolus within the nucleus [79]. (**D**) The strain JL408 (*MAT*α, *hog*1Δ; P_*GPD1*_-mCherry-*CRZ1*) was generated from a cross between the *hog1*Δ mutant in the mating type **a** background and the strain JL410 (*MAT*α, P_*GPD1*_-mCherry-*CRZ1*). JL408 was cultured in YP or YP+GlcN medium overnight at 22°C. (**E**) The wild-type strain H99 was grown to mid-logarithmic phase and then exposed to 1 M NaCl or 2% glucosamine (YPGlcN) for the indicated time points. Hog1 phosphorylation levels were monitored using anti-P-p38 antibody. The blot was stripped and then used for detection of Hog1 protein level with a polyclonal anti-Hog1 antibody as a loading control.

Multiple components of the HOG pathway, namely Tco1 (hybrid histidine kinase), Ssk1 (response regulator), Ssk2 (MAPKKK), and Pbs2 (MAPKK), suppress glucosamine-stimulated filamentation given that disruption of these components enhanced filamentation (Fig 3). We decided to examine if the HOG pathway components suppress filamentation through Crz1. For this purpose, we made double gene deletion mutants *ssk*2Δ *crz1*Δ and *pbs*2Δ* crz1*Δ and examined their phenotypes on glucosamine medium. The *ssk*2Δ and *pbs*2Δ single mutants showed enhanced filamentation on glucosamine medium (Fig 3, Fig 8A and 8B). The *ssk*2Δ* crz1*Δ and *pbs*2Δ *crz1*Δ double mutants were non-filamentous on glucosamine medium (Fig 8A), similar to the *crz1*Δ single mutant. This result suggests that Crz1 is essential in the regulation of filamentation in response to glucosamine and it functions downstream of Ssk2 and Pbs2.

We postulate that the HOG pathway components may oppose the effect of calcineurin and suppress the nuclear translocation of Crz1. If this hypothesis is valid, then disruption of the HOG pathway components would increase the level of nucleus-localized Crz1. To test this hypothesis, we constructed mCherry labeled Crz1 in the *ssk1*Δ mutant, the *ssk2*Δ mutant, and the *pbs2*Δ mutant by crossing these strains to XW252 (P_*CRZ1*_-Crz1-mCherry, GFP-Nop1) [79]. We then examined the subcellular localization of Crz1-mCherry in the absence of these HOG pathway components. We found that most cells showed nuclear localized Crz1-mCherry next to the nucleolus marker GFP-Nop1 in the absence of Ssk1, Ssk2, or Pbs2 even when these cells were cultured in YPD medium at 22°C without any stimulus (Fig 8C). Upon induction with glucosamine, almost all cells showed nuclear localized Crz1, regardless whether the *SSK1*, *SSK2*, or *PBS2* gene was intact or not (Fig 8C). Thus, the absence of the HOG pathway upstream components increased the basal level of nuclear localized Crz1, which may have enhanced the initiation of filamentation in the *ssk1*Δ, *ssk2*Δ, or *pbs2*Δ mutant on glucosamine medium. In contrast to the deletion of *SSK1*, *SSK2*, or *PBS2*, the deletion of *HOG1* gene did not significantly enhance the basal level of nuclear-translocation in the absence of glucosamine compared to the wild type (generated by crossing P_*GPD1*_-mCherry-*CRZ1* to *hog1*Δ) (left panel in Fig 8D). Nonetheless, the treatment of glucosamine stimulated the translocation of cytosolic Crz1 into the nucleus in the *hog1*Δ strain, just like the wild type (right panel in Fig 8D). Thus, Hog1, the downstream MAPK of the HOG pathway, appears to be dispensable for glucosamine-stimulated filamentation. In the wild-type H99 strain, Hog1 is known to be highly phosphorylated under normal growth conditions and it undergoes dephosphorylation in response to osmotic shock [81]. Indeed, we observed reduced level of phosphorylation of Hog1 in response to osmotic stress caused by NaCl (Fig 8E). However, no apparent change in Hog1 phosphorylation was observed in response to glucosamine (Fig 8E). This result indicates that Hog1 phosphorylation is not affected by glucosamine, which corroborates the dispensability of Hog1 in glucosamine-stimulated filamentation.

## Discussion

*C*. *neoformans* could undergo yeast-to-hypha transition and this morphotype switch is linked to its virulence potential. Yeast is the virulent form, whereas the filamentous form is attenuated in virulence in mammalian models of cryptococcosis ([82] and references therein). Our previous studies demonstrated that upregulation of *ZNF2* is sufficient to drive *C*. *neoformans* to undergo filamentation and to abolish/attenuate virulence [24–26]. Thus, activation of filamentation could potentially be used to mitigate cryptococcosis if suitable effectors that can trigger cryptococcal filamentation program *in vivo* can be identified. In addition, the filamentous form of *Cryptococcus* elicits protective immune-responses in a mammalian host [26], providing a platform for future vaccine development.

Because the pheromone pathway has no or minimal impact on virulence and *C*. *neoformans* infections are largely caused by serotype A α isolates (α >99% among serotype A isolates), it is of great value to identify conserved signals and pathways that control self-filamentation independent of the pheromone pathway. Self-filamentation in *C*. *neoformans* is mostly observed in serotype D isolates and rarely in serotype A isolates. The widely used and highly virulent serotype A reference strain H99, for instance, has not been observed to undergo self-filamentation under laboratory conditions despite numerous attempts. Here we found that H99 can undergo self-filamentation in response to glucosamine and this morphological transition is independent of the pheromone pathway. Why glucosamine, but not any other carbon-source tested, triggers self-filamentation in H99 remains mysterious. Glucosamine is the subunit of chitosan from *Cryptococcus* cell wall. Chitosan is the deacetylated form of chitin, and chitin is a common cell wall component in fungi and in the exoskeletons of arthropods, such as the shells of crustaceans and the outer coverings of insects. It is possible that the presence of glucosamine, rather than N-acetyl glucosamine, the subunit of chitin, serves as a unique danger signal to *Cryptococcus*. Alternatively, unknown secondary signals triggered by glucosamine are the real signals stimuli of filamentation. Regardless of the true biological meaning of glucosamine, the identification of pathways that control self-filamentation in natural serotype A strains like H99 represents an important advance in the endeavors to understand the regulation of cryptococcal dimorphism, which was primarily considered a response to pheromone.

**Fig 9:**
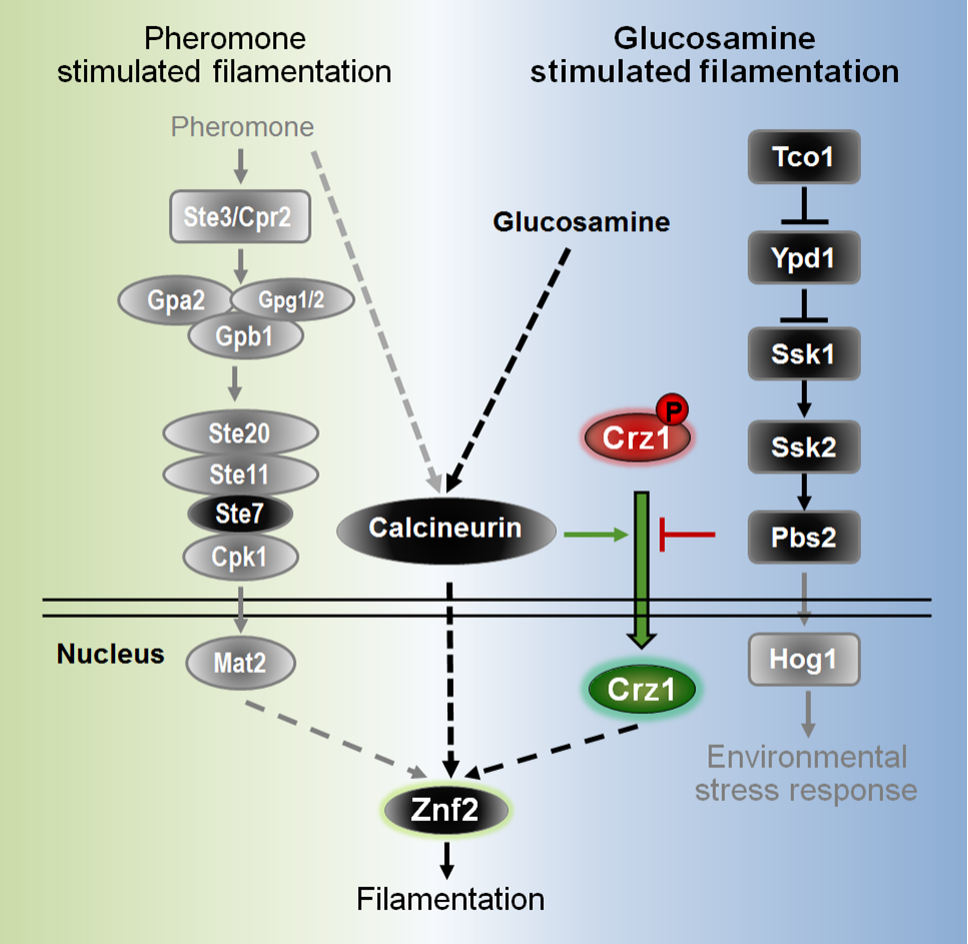
Proposed model of genetic regulation of glucosamine-stimulated filamentation in *Cryptococcus neoformans*.

By screening approximately 2500 gene deletion mutants for altered filamentation on glucosamine medium, we found that the transcription factor Crz1 was critical for glucosamine-induced filamentation: deletion of *CRZ1* abolished filamentation and overexpression of *CRZ1* enhanced filamentation on glucosamine medium. Crz1 appears to regulate filamentation specifically in response to glucosamine, as neither deletion nor overexpression of *CRZ1* showed any effect on cryptococcal yeast-to-hypha transition during mating on V8 medium (S6 Fig)[38]. We found that the pheromone pathway responding to mating cues was overall dispensable for filamentation in response to glucosamine (Fig 2). Glucosamine strongly induced Crz1 to translocate from the cytosol to the nucleus, where it can exert its function as a transcription factor. Two pathways converged on Crz1 and play important but opposing roles. One pathway is the expected calcineurin pathway known to dephosphorylate Crz1, which required for its nuclear translocation [75, 79]. Indeed, Crz1 was retained in the cytoplasm in the absence of calcineurin catalytic subunit Cna1 or the regulatory subunit Cnb1 (Fig 7B and 7C). Interestingly, the absence of Cbp1 didn’t affect Crz1’s translocation into the nucleus (Fig 7C). This offers a plausible explanation for the lack of dramatic phenotype of the *cbp1*Δ mutant, in contrast to the non-filamentous phenotype of the *cna1*Δ and the *cnb1*Δ mutant on glucosamine medium (Fig 4B). The other pathway is the HOG components upstream of the Hog1 MAPK, which is known for their regulation of a variety of environmental stress responses. We found that the HOG components inhibited filamentation on glucosamine medium and suppressed the nuclear translocation of Crz1 (Fig 8C), likely through their direct or indirect effect on Crz1 phosphorylation that counter-balances the phosphatase activity of calcineurin (Fig 9).

In another fungal pathogen *Aspergillus fumigatus*, CrzA (Crz1 homolog) translocates to the nucleus upon osmotic stress caused by NaCl or sorbitol [83]. CrzA also directly upregulates the expression of the histidine kinase PhkB and the MAPKKK SskB of the osmotic sensing pathway by binding to their promoters [83]. Thus in *A*. *fumigatus*, CrzA plays a role in osmotic stress response [83], and there appears to be a positive feedback regulation between the osmotic sensing pathway and CrzA in *A*. *fumigatus*. Unlike CrzA in *A*. *fumigatus*, Crz1 in *Cryptococcus* translocates to granule-like structures in the cytoplasm after osmotic stress [46] (Fig 6). Consistent with its cytoplasmic localization in response to osmotic stress, the *crz1*Δ mutant was as resistant to the osmotic stress caused by NaCl as the wild type (S8 Fig). Overexpression of *CRZ1* in the *pbs2*Δ mutant also failed to restore *pbs2*Δ’s sensitivity to osmotic stress (S8 Fig). These findings are consistent with the idea that Crz1 is not critical for the osmotic stress response in *C*. *neoformans* (S8 Fig).

The calcineurin pathway is known to control growth, stress responses, morphogenesis and pathogenicity in various fungal species [70–72, 78, 84–90]. However, Crz1, the established downstream target of calcineurin, appears to be more specific in promoting hyphal growth than the adaptation to the general stresses based on previous studies in *A*. *fumigatus* [91, 92] and *Candida* species [85, 93]. Interestingly, the HOG pathway plays a more suppressive role in hyphal growth as demonstrated in *Candida* species [94–96]) and in *Cryptococcus neoformans* during bisexual mating [69, 81]. Thus the opposing effect between the calcineurin pathway and the HOG pathway on hyphal growth might be conserved in multiple fungal species. Whether Crz1 is the conserved conjunction of these two pathways in regulating filamentation in these fungal species is yet to be determined.

We believe that the upstream components of the HOG pathway normally suppress the translocation of Crz1 to the nucleus based on the elevated basal level of nuclear Crz1 in the corresponding deletion mutants in the absence of any stimuli (Fig 8C). This suggests that the upstream components of the HOG pathway inactivate Crz1, possibly by enabling the phosphorylation of Crz1 either directly or indirectly, and consequently opposing the activity of calcineurin. It is important to note that nuclear localization of Crz1 is necessary, but not sufficient to drive filamentation in the absence of glucosamine. This is evident given that some cells showed nuclear localized Crz1 even in YPD medium, but all cells grew in the yeast form under that condition. This is also consistent with the observation that heat-shock and calcium, although both stimulate nuclear translocation of Crz1, were unable to elicit filamentation in H99 in the absence of glucosamine. Thus, a yet unknown factor affected by glucosamine, in addition to the requirement of Crz1 nuclear translocation, has to be involved to enable filamentation.

One interesting observation is that not all components in a well-established pathway behave in the same fashion. For example, most key components in the pheromone pathway, including the transcription factor Mat2, are dispensable for self-filamentation induced by glucosamine. However, *ste7*Δ is non-filamentous on glucosamine medium. This finding is surprising given that *ste7*Δ and *mat2*Δ are both non-filamentous with identical transcriptomes under mating-inducing conditions during both bisexual and unisexual development [25, 32, 97]. Thus, the distinct phenotype of *ste7*Δ on glucosamine medium suggests that Ste7 might have additional functions besides its established role in pheromone sensing and response. Another example is Hog1 in the HOG pathway. Most upstream components of the HOG pathway suppress filamentation on glucosamine medium, but the MAPK Hog1 itself shows no or minimal involvement in this process. We postulate that there is a divergence in the downstream effectors of this phosphorelay system in response to osmotic stress or glucosamine. Hog1 is activated in response to osmotic stress when Crz1 is being concentrated in granules in the cytoplasm in *Cryptococcus* [75] (Fig 6A). In contrast, Crz1 is localized to the nucleus in response to glucosamine. How different effectors are activated by the same phosphorelay system, what controls the multiple distinct subcellular localizations of Crz1, and what prevents cross-activation of the downstream effectors remain to be investigated.

## Materials and methods

### Media and growth conditions

Strains were stored as glycerol stocks in -80°C. Freshly streaked cells were used for experiments. The three deletion sets made in the H99 background were obtained from the Fungal Genetics Stock Center (FGSC) and the information about these strains can be obtained from the FGSC website (http://www.fgsc.net/crypto/crypto.htm). Other strains were listed in S2 Table. Cryptococcal cells were maintained on YPD medium (20 peptone, 10 yeast extract, 20 glucose, 20 agar, gram/liter) unless stated otherwise.

### Filamentation assay

For the filamentation assay, the YP medium (20 peptone, 10 yeast extract, 20 agar, gram/liter) was used as the base medium. All the different carbon sources tested were made to the final concentration of 2%. When testing filamentation on YPGlcN medium (20 peptone, 10 yeast extract, 20 glucosamine, 20 agar, gram/liter), 3 μl of cells (optical density OD_600_ = 1) of the tested strains were dropped onto the agar medium. Cells were cultured at 30°C for two days before being transferred to 22°C for additional incubation of 4 to 7 days in the dark. To test the effect of the calcineurin inhibitor FK506 on filamentation, FK506 was added to the YPGlcN medium at the final concentration of 1 μg/ml. To test the dose-dependent effects of glucosamine on filamentation, glucosamine were added to the YP base medium to the final concentration of 0, 0.2%, 0.5%, 1%, and 2%. To test the effect of the addition of another carbon source to the YPGlcN medium on filamentation, 2% galactose, glycerol, or xylose was added to the YPGlcN medium (2% glucosamine).

### Phenotypical assays

To test thermo-tolerance, cells of the tested strains with 5x serial dilutions (OD_600_ = 10, 2, 0.4, 0.08, 0.016, and 0.0032) were dropped onto YPD medium and incubated at 30°C or 37°C for 2 days. To test the susceptibility to cell wall stress, cells of the indicated strains were serial diluted and spotted onto YPD medium, YPD with 0.2% Congo Red, or YPD medium with 10 μg/ml of Calcofluor white. Cells were then incubated at 30°C for 2 days.

### RNA extraction and qPCR

RNA extraction and qPCR were performed as we described previously [24]. For the transcript measurements used in Fig 2, strains H99, *mat2*Δ, and *znf2*Δ were cultured on YPD medium or glucosamine medium at 30°C for 2 days, and then were transferred to 22°C for additional 2 days before cells were harvested. For the transcript measurements used in Fig 5, Strains H99, *crz1*Δ, and *znf2*Δ were cultured on the YP-glucosamine or YP base medium at 30°C for 2 days, and then incubated at 22°C for additional incubation. Cells were harvested at the time points (0, 2 days, 4 days, and 6 days) as indicated in the figures.

Harvested cells were washed with cold water, frozen in liquid nitrogen, and then lyophilized. Lyophilized cells were broken into fine powder with glass beads and total RNA was extracted with the PureLink RNA Mini Kit (life technology) according to the manufacture’s instruction. First strand cDNA was synthesized with Superscript III cDNA synthesis kit (Invitrogen) according to the manufacture’s instruction. The house-keeping gene *TEF1* was used as the endogenous control. The relative transcript levels were determined using the comparative ΔΔCt method as described previously [24]. Three biological replicates were performed for each sample and their values were used to calculate the mean and the standard error. Primers used for realtime PCR were listed in S3 Table.

### Genetic screen of the gene deletion mutants on glucosamine medium

All the gene deletion mutants in the serotype A background used in this study generated by the Lin’s group or Bahn’s group were made in the same H99 background (see S2 Table for strains used in this study). The 2015 gene deletion set deposited by Dr. Hiten Madhani’s lab and the transcription factor and kinase gene deletion sets deposited by Dr. Bahn’s lab are available from the Fungal Genetics Stock Center. http://www.fgsc.net/crypto/crypto.htm). These mutants were also generated in the same H99 background. The mutants were screened on the YP-glucosamine (2%) medium after replicating from 96 well plates as described earlier for the filamentation assays. Strains with altered filamentation were selected based on comparison with other strains on the same plate during the initial screen. These mutant phenotypes were further confirmed in the secondary screen with the wild type H99 control. For the genes and pathways that were further characterized in this study, including the pheromone pathway, the calcineurin pathway, and the Hog1 pathway, separate mutants were obtained from the original sources where the mutations were verified in the previously published work. These strains and their sources/references were listed in S2 Table.

### Gene deletion and gene overexpression

To generate the knockout construct, 1 kb of the 5’ and 3’ flanking sequences bordering the open reading frame of the gene of interest were amplified using the genomic DNA of the wild-type strain as the template. They were then fused with the NEO or NAT dominant drug marker amplified from the plasmid pAI1 or pJAF1 by overlap PCR as we described previously [98]. The knockout constructs were introduced into appropriate recipient strains by biolistic transformation as described previously [99]. The transformants grown on selective medium (YPD+NAT or YPD+G418/NEO) were then screened for gene replacement *via* homologous recombination events by diagnostic PCR as described previously [98]. To generate *CRZ1* or *ZNF2* overexpression strains, the open reading frame of the *CRZ1* or the *ZNF2* gene were first amplified by PCR with specifically designed primers with FseI/PacI cut sites at the ends. After digestion, the digested products were ligated into the P_*GPD1*_ vector or the P_*CTR4*_ vector where the ORF was placed downstream of the *GPD1* or the *CTR4* promoter, as we described previously [58, 59]. The resulting plasmids were then linearized and introduced into the recipient strains as indicated in the text by biolistic transformation. All the primers used for constructing or confirming gene deletion or gene overexpression were listed in S3 Table.

### Mating and genetic crosses

Yeast cells of α and **a** mating partners were mixed together on V8 juice agar medium (5% V8 juice, 0.5 g/L KH_2_PO_4_, 4% agar, pH adjusted to 5). The mixed culture was then incubated for 2 weeks at 22°C in the dark until spores were produced following filamentation. Cells from V8 medium were transferred to fresh YPD agar medium and spores were micro-manipulated with a dissecting microscope. The mating type of the germinated spores was determined by successful mating of their derived colonies with either JEC20**a** or JEC21α. Genetic linkage between the presence of the drug marker and the observed mutant phenotype was established by analyzing the dissected spores as we described previously [42].

### Protein tagging

To characterize the subcellular localization of Znf2 and Crz1, mCherry was fused to the N-terminus of Znf2 or Crz1 in frame. The ORF of *CRZ1* or *ZNF2* with *Pac*I recognition site at 3’ end was amplified by PCR and then fused at the N-terminus with mCherry carrying *Fse*I recognition site at its 5’end. The fragment mCherry-*CRZ1* and mCherry-*ZNF2* was digested with FseI and PacI and then ligated into the P_*GPD1*_ vector or the P_*CTR4*_ vector. The construct of the mCherry tagged protein controlled by the *GPD1* or the *CTR4* promoter (P_*GPD1*_-mCherry-*CRZ1* and P_*CTR4*_-mCherry-*ZNF2*) were then introduced into the recipient strains by biolistic transformation as described previously [99]. The N-terminal tagged Crz1 and Znf2 are functional based on the observation that they could restore the filamentation defect observed in the corresponding gene deletion mutants.

### Microscopic examination

Colony morphology was examined with a SZX16 stereoscope (Olympus). Colony images were captured with a GO-21camera and acquired using the QIMAGINE software. To determine the subcellular localization of mCherry-Crz1 or mCherry-Znf2, cells were observed with a Zeiss M2 epi-fluorescence microscope and images were acquired with the AxioCam MRm camera and processed with the software Zen 11 (Carl Zeiss Microscopy). The filter used for visualizing mCherry was the FL filter set 43 HE cy3 (Carl Zeiss Microscopy). GFP was visualized using the filter FL filter set 38 HE GFP (Carl Zeiss Microscopy). To visualize the nuclei, cells were fixed in a fixer solution (3.7% formaldehyde; 1X PBS; 1% Triton X) for 10 min and then stained with DAPI (0.4 μg/ml) for 15 min. The filter used to visualizing DAPI was FL Filter Set 49 DAPI (Carl Zeiss Microscopy).

### Crz1-mCherry translocation assay

To examine the effect of temperature on Crz1 localization, cells of the tested strains were cultured in the YPD medium at 22°C, 30°C, or 37°C overnight. To test the effects of different conditions or mutations on the subcellular localization of mCherry tagged Crz1, *Cryptococcus* cells were grown in liquid media at 22°C for 10 hours. To examine the impact of glucosamine on Crz1’s subcellular localization, cells were cultured in the YP-glucosamine (2%) liquid medium at 22°C for 10 hrs. To examine the impact of calcium or salt on the localization of Crz1, cells were cultured first in YPD at 22°C, centrifuged, washed with PBS, and then suspended in 100 mM CaCl_2_ or 1.5 M NaCl for 10–30 minutes. To test the impact on Crz1’s subcellular localization by the deletion of *SSK1*, *SSK2*, *PBS2*, *CNA1*, *CNB1* or *CBP1*, the corresponding *Cryptococcus* strains were cultured in either YPD or glucosamine medium at 22°C for 10 hours. To quantify the percentage of cells with Crz1 localized to the nucleus, the numbers of cells with Crz1 in the nucleus and the total cells with fluorescence signals were determined and the ratio was calculated in three replicated samples. The data were used to calculate the mean value of the population with nuclear localization and the standard errors.

### Western blot analysis of Hog1 phosphorylation

The overnight culture of wild-type strain H99 was inoculated in fresh YPD liquid medium (250 ml) and incubated at 30°C until the culture reached the optical density of approximately 0.9–1.0 at 600 nm (OD_600_). Cells were harvested by centrifugation, washed two times in PBS, and resuspended in YPD medium containing 1 M NaCl or in YP medium containing 2% glucosamine. At each designated time point, an aliquot of 50 ml of the culture was mixed with an equal volume of ice-cold stop solution (0.9% NaCl, 1 mM NaN_3_, 10 mM EDTA, 50 mM NaF). The cells were then collected by centrifugation and resuspended in lysis buffer [50 mM Tris-Cl (pH 7.5), 1% sodium deoxycholate, 5 mM sodium pyrophosphate, 0.2 mM sodium orthovanadate, 50 mM NaF, 0.1% SDS, 1% Triton X-100, 0.5 mM phenylmethylsulfonyl fluoride, and 2.5× protease inhibitor cocktail solution (Calbiochem)]. The resuspended cells were disrupted using a bead-beater for 6 cycles (30 sec bead beating with 2 min rest intervals). Protein concentrations were determined with the Pierce BCA protein assay kit (Thermo Fisher Scientific). A total of 5 μg of proteins were loaded into 10% SDS-polyacrylamide gel and analyzed by western blot using a primary antibody of rabbit P-p38 MAPK specific antibody (Cell Signalling Technology) to detect phosphorylated Hog1 and polyclonal anti-Hog1 antibody (Santa Cruz) for the detection of Hog1 as a loading control. Anti-rabbit IgG horseradish peroxidase-conjugated antibody (Santa Cruz) was used as a secondary antibody. The blot was developed using the ECL western blotting detection system according to the instruction of the manufacture (Bio-Rad).

### Statistical analysis

Statistical significance of different groups in terms of Crz1 localization was assessed by the *t*-test. The statistical analyses were performed using the Graphpad Prism 5 program, with *p* values lower than 0.05 considered statistically significant.

## Supporting information

S1 Fig Phenotypes of *Candida albicans* strains on YPD, YP+GlcNAc, YP+GlcN, and YP+Deoxyl-Glc. Cells (optical density of OD_600_ = 1.0) were dropped onto the indicated medium and cultured at 30°C for 2 days followed by additional incubation at 22°C for 4 days.
(PDF)

S2 Fig Znf2, but not Mat2, is required for filamentation of the serotype D strain XL280 on glucosamine medium. The wild-type serotype D strain XL280 and the corresponding *mat2*Δ and *znf2*Δ mutants were cultured on V8 juice agar medium for 6 days at 22°C or on glucosamine medium for 2 days at 30°C followed by additional 4 days of incubation at 22°C. (upper two panels). The wild-type serotype A strain H99 and the corresponding *mat2*Δ and *znf2*Δ mutants were cultured on V8 juice agar medium at 22°C for 4 days.
(PDF)

S3 Fig Phenotypes of the hexamine metabolism mutants *gnd1*Δ and *gnat1*Δ. (**A**) A diagram of the hexamine metabolism pathway. HK: Hexose Kinase, GNAD: Glucosamine Deacetylase, GND: Glucosamine Deaminase, GNAT: Glucosamine N-acetyl transferase. (**B**) The growth of the *gnd1*Δ mutant is hypersensitive to glucosamine. Wild-type H99 and the *gnd1*Δ mutant were cultured at 30°C for 2 days on YP medium containing glucosamine of different concentrations with or without the addition of other carbon sources (galactose, glycerol, or xylose). (**C**) The *gnd1*Δ mutant filamented similarly as the wild-type strain H99. The wild-type H99 and the *gnd1*Δ mutant were cultured on glucosamine medium for 2 days at 30°C followed by additional 4 days of incubation at 22°C.
(PDF)

S4 Fig Colony images of represented strains of the four classified groups based on the robustness of filamentation on glucosamine medium compared to the control. (PDF)

S5 Fig Mutants in the calcineurin pathway showed different susceptibility towards various stresses. Cells from the wild-type H99, the *cna1*Δ mutant, the *cnb1*Δ mutant, the *cbp1*Δ mutant, and the *crz1*Δ mutant were serial diluted (5x) and spotted onto YPD medium or YPD medium with Calcofluor white/CFW (10 μg/ml) or Congo Red (0.2%). The cells on YPD medium were incubated at 30°C or 37°C as indicated. Cells on medium with Calcofluor white or Congo red were cultured at 30°C.
(PDF)

S6 Fig Crz1 does not regulate pheromone-induced filamentation. (**A**) The wild-type H99, the *crz1*Δ mutant, and the *CRZ1*^*oe*^ strain all in the mating type α were cultured alone on V8 juice agar medium at 22°C for 9 days. 3 μl of cells at the density of OD_600_ = 3 were used to inoculate. (**B**) The wild-type H99α, the *crz1*Δ α mutant, and the *CRZ1*^*oe*^ α strain were mixed with the mating partner KN99**a** of the opposite mating type. The mixed co-cultures were inoculated and cultured on V8 medium at 22°C in the dark for 9 days.
(PDF)

S7 Fig Calcium, high temperature, and glucosamine induce the nuclear translocation of Crz1-mCherry expressed under its native promoter. To test temperature’s effect on the subcellular localization of Crz1-mCherry, the strain XW252 (P_*CRZ1*_- *CRZ1*-mCherry, GFP-Nop1) was cultured in YPD liquid at 37°C with shaking for 9 hours. To test the effect of calcium, cells of the strain P _*CRZ1*_-mCherry-*CRZ1* were first collected from the culture in liquid YPD at 22°C for 9 hours and then suspended in YPD with 100 mM of CaCl_2_ for 10–20 min. To test the effect of glucosamine, the strain P_*CRZ1*_-mCherry-*CRZ1* was cultured in YP-glucosamine liquid medium at 22°C for 9 hours. For the examination of a shift in carbon-source, cells were incubated in YP-glucosamine liquid medium at 22°C for 9 hours and then shifted to glucose medium at 22°C for 90 minutes. (**A**) Images of the cells under the conditions tested. (**B**) Quantification of cells with nuclear localization of Crz1.
(PDF)

S8 Fig Crz1 is not important for osmotic stress response. Cells of the following strains (wild-type H99, *crz1*Δ, *cna1*Δ, *cna1*Δ*CRZ1*^*oe*^, *CRZ1*^*oe*^, *pbs2*Δ, *pbs2*Δ*crz1*Δ, and *pbs2*Δ*CRZ1*^*oe*^) were serial diluted and spotted onto YPD medium with or without the addition of NaCl at 1M, or 1.5M. Cells were then incubated at 30°C or 37°C for 3 days before images were taken.
(PDF)

S1 Table The screening results from forward genetic screening. (DOCX)

S2 Table The strains used in this research. (DOCX)

S3 Table The primers used in this research. (DOCX)
