# Supplementary figures and images for: Glucosamine stimulates pheromone-independent dimorphic transition in Cryptococcus neoformans by promoting Crz1 nuclear translocation
Source: PLoS Genet. 2017 Sep 12;13(9):e1006982. doi: 10.1371/journal.pgen.1006982 (PMC5595294; doi:10.1371/journal.pgen.1006982)

Supplemental Figure 1

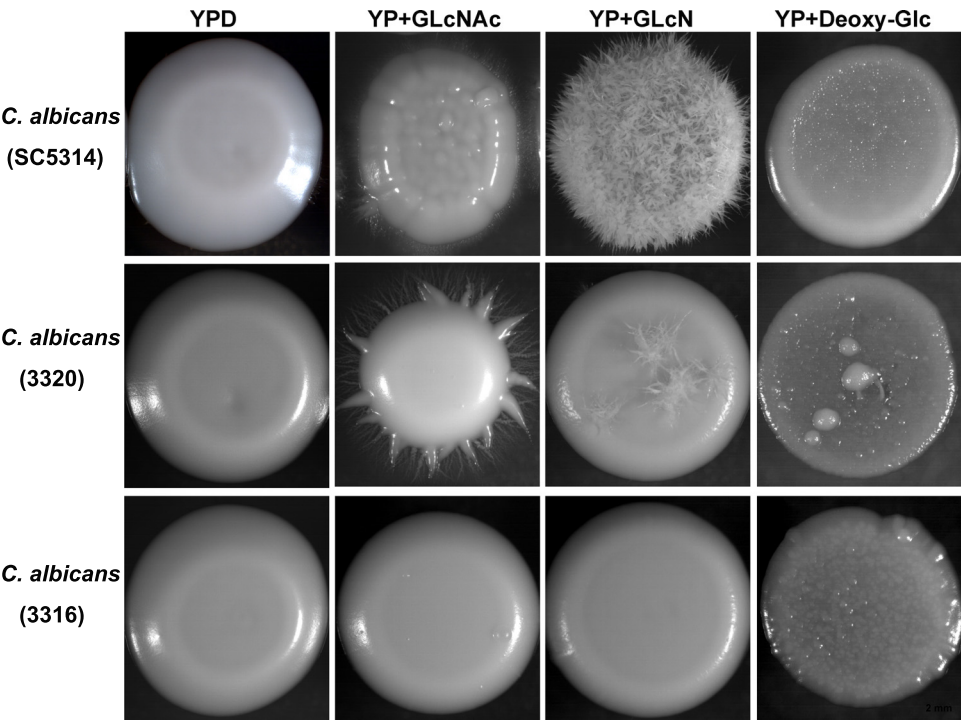

Supplement: S1 Fig — Cells (optical density of OD600 = 1.0) were dropped onto the indicated medium and cultured at 30°C for 2 days followed by additional incubation at 22°C for 4 days. (PDF) [file pgen.1006982.s001.pdf]

Supplemental Figure 2

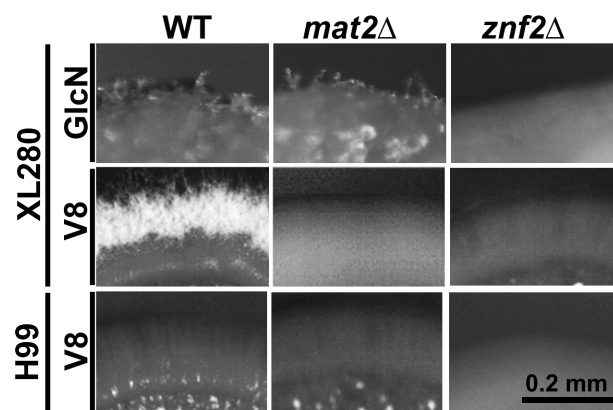

Supplement: S2 Fig — The wild-type serotype D strain XL280 and the corresponding mat2Δ and znf2Δ mutants were cultured on V8 juice agar medium for 6 days at 22°C or on glucosamine medium for 2 days at 30°C followed by additional 4 days of incubation at 22°C. (upper two panels). The wild-type serotype A strain H99 and the corresponding mat2Δ and znf2Δ mutants were cultured on V8 juice agar medium at 22°C for 4 days. (PDF) [file pgen.1006982.s002.pdf]

Supplemental Figure 3

**A**

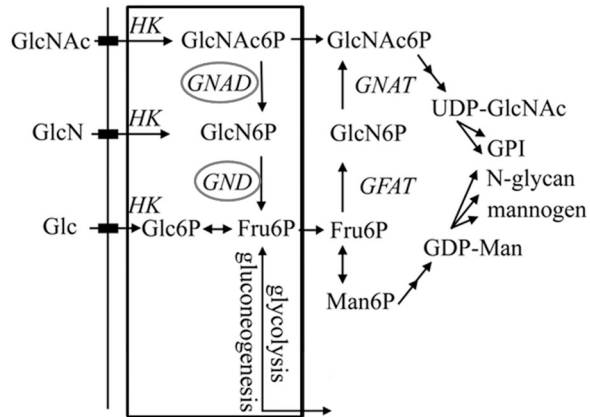

**B**

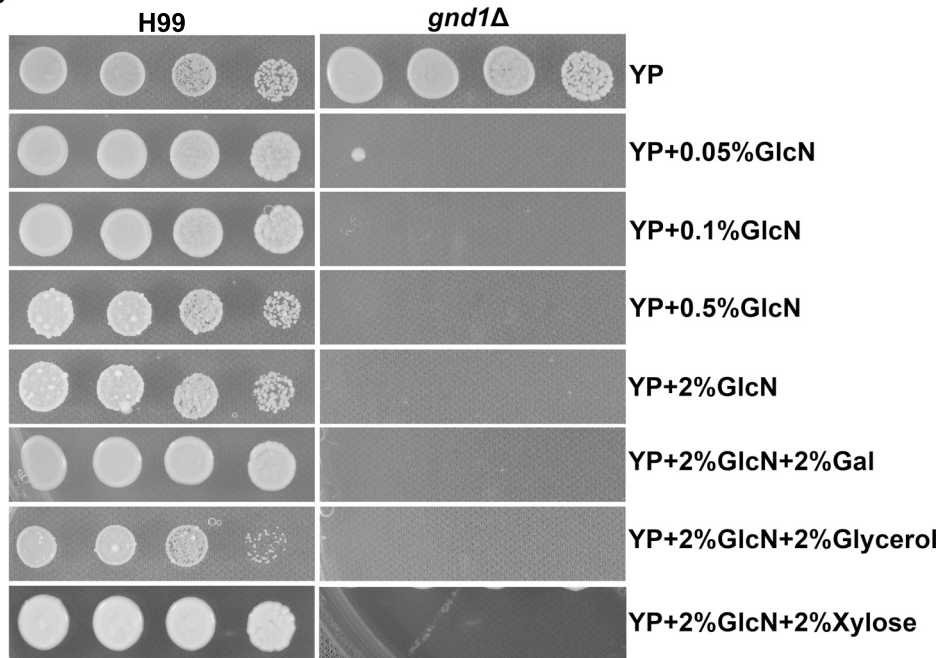

**C**

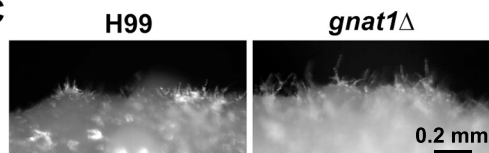

Supplement: S3 Fig — (A) A diagram of the hexamine metabolism pathway. HK: Hexose Kinase, GNAD: Glucosamine Deacetylase, GND: Glucosamine Deaminase, GNAT: Glucosamine N-acetyl transferase. (B) The growth of the gnd1Δ mutant is hypersensitive to glucosamine. Wild-type H99 and the gnd1Δ mutant were cultured at 30°C for 2 days on YP medium containing glucosamine of different concentrations with or without the addition of other carbon sources (galactose, glycerol, or xylose). (C) The gnd1Δ mutant filamented similarly as the wild-type strain H99. The wild-type H99 and the gnd1Δ mutant were cultured on glucosamine medium for 2 days at 30°C followed by additional 4 days of incubation at 22°C. (PDF) [file pgen.1006982.s003.pdf]

Supplemental Figure 4

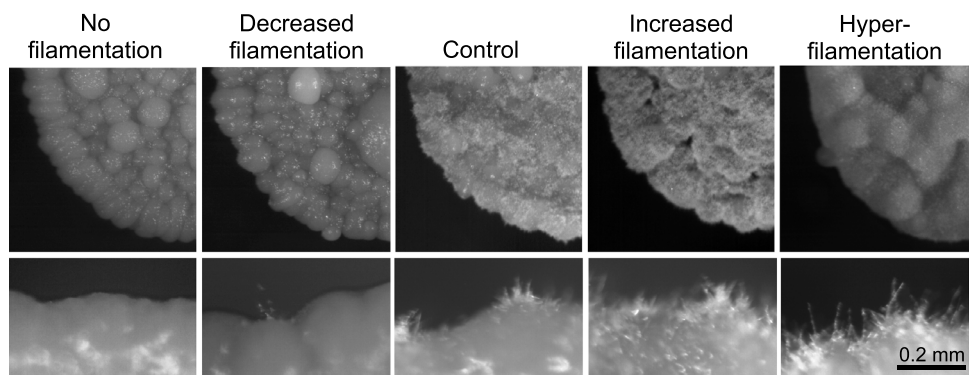

Supplement: S4 Fig — (PDF) [file pgen.1006982.s004.pdf]

Supplemental Figure 5

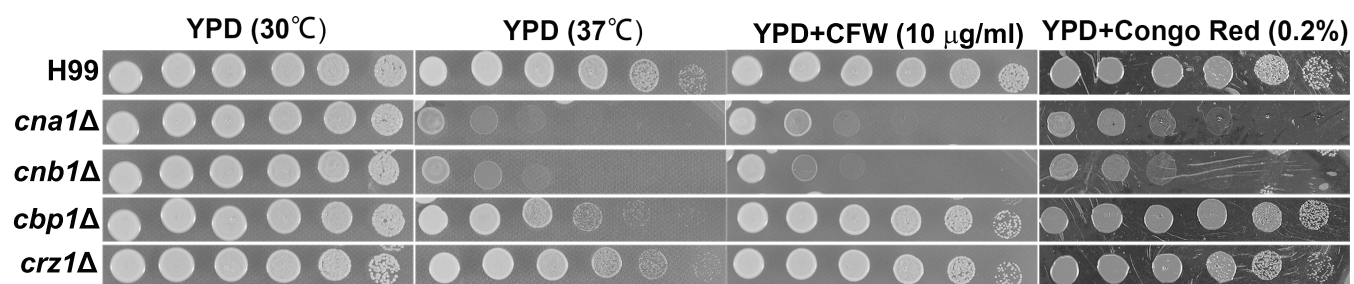

Supplement: S5 Fig — Cells from the wild-type H99, the cna1Δ mutant, the cnb1Δ mutant, the cbp1Δ mutant, and the crz1Δ mutant were serial diluted (5x) and spotted onto YPD medium or YPD medium with Calcofluor white/CFW (10 μg/ml) or Congo Red (0.2%). The cells on YPD medium were incubated at 30°C or 37°C as indicated. Cells on medium with Calcofluor white or Congo red were cultured at 30°C. (PDF) [file pgen.1006982.s005.pdf]

Supplemental Figure 6

**A**  $\alpha$  alone on V8

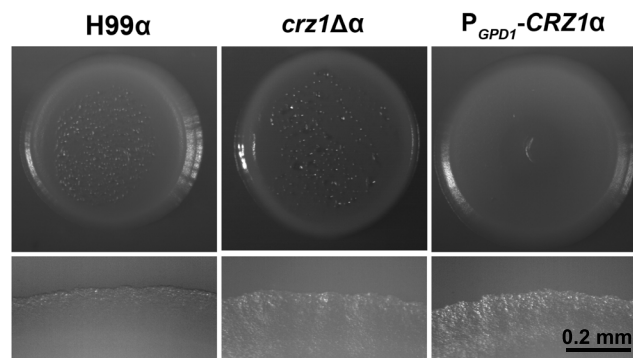

**B**  $\alpha$  and a coculture on V8

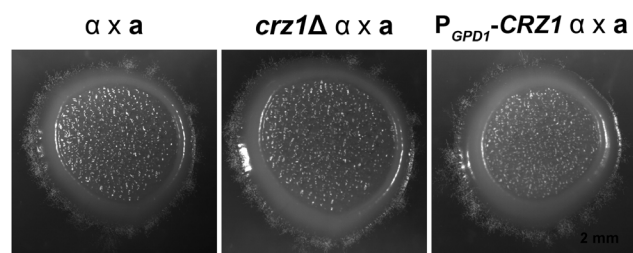

Supplement: S6 Fig — (A) The wild-type H99, the crz1Δ mutant, and the CRZ1oe strain all in the mating type α were cultured alone on V8 juice agar medium at 22°C for 9 days. 3 μl of cells at the density of OD600 = 3 were used to inoculate. (B) The wild-type H99α, the crz1Δ α mutant, and the CRZ1oe α strain were mixed with the mating partner KN99a of the opposite mating type. The mixed co-cultures were inoculated and cultured on V8 medium at 22°C in the dark for 9 days. (PDF) [file pgen.1006982.s006.pdf]

Supplemental Figure 7

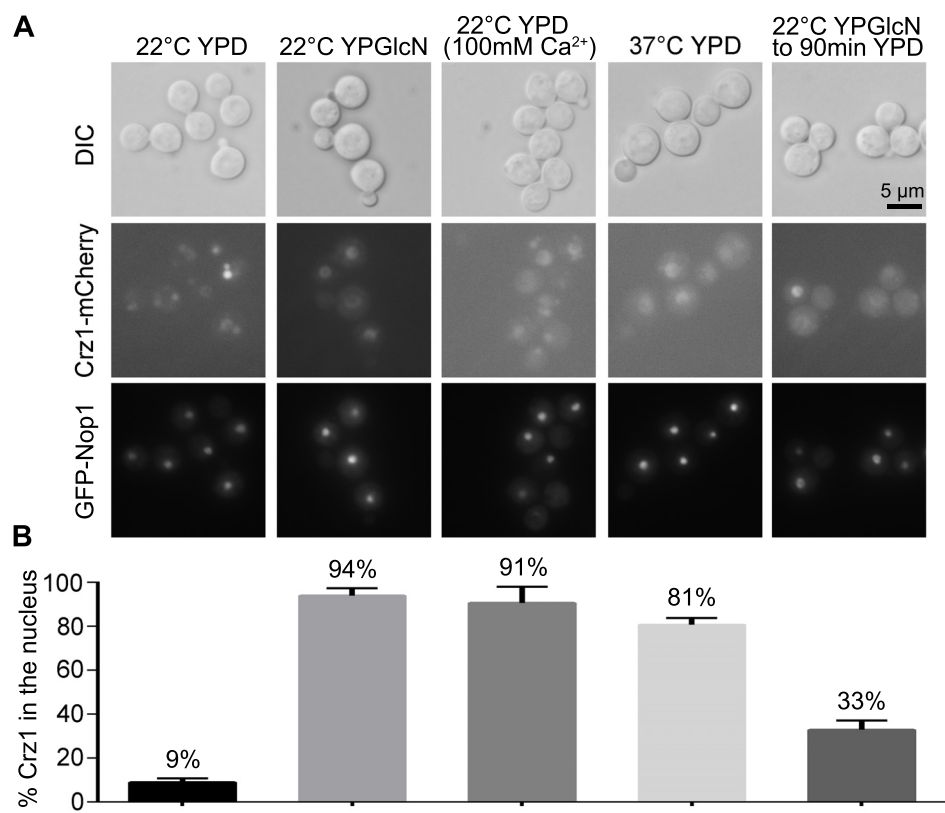

Supplement: S7 Fig — To test temperature’s effect on the subcellular localization of Crz1-mCherry, the strain XW252 (PCRZ1- CRZ1-mCherry, GFP-Nop1) was cultured in YPD liquid at 37°C with shaking for 9 hours. To test the effect of calcium, cells of the strain P CRZ1-mCherry-CRZ1 were first collected from the culture in liquid YPD at 22°C for 9 hours and then suspended in YPD with 100 mM of CaCl2 for 10–20 min. To test the effect of glucosamine, the strain PCRZ1-mCherry-CRZ1 was cultured in YP-glucosamine liquid medium at 22°C for 9 hours. For the examination of a shift in carbon-source, cells were incubated in YP-glucosamine liquid medium at 22°C for 9 hours and then shifted to glucose medium at 22°C for 90 minutes. (A) Images of the cells under the conditions tested. (B) Quantification of cells with nuclear localization of Crz1. (PDF) [file pgen.1006982.s007.pdf]

Supplemental Figure 8

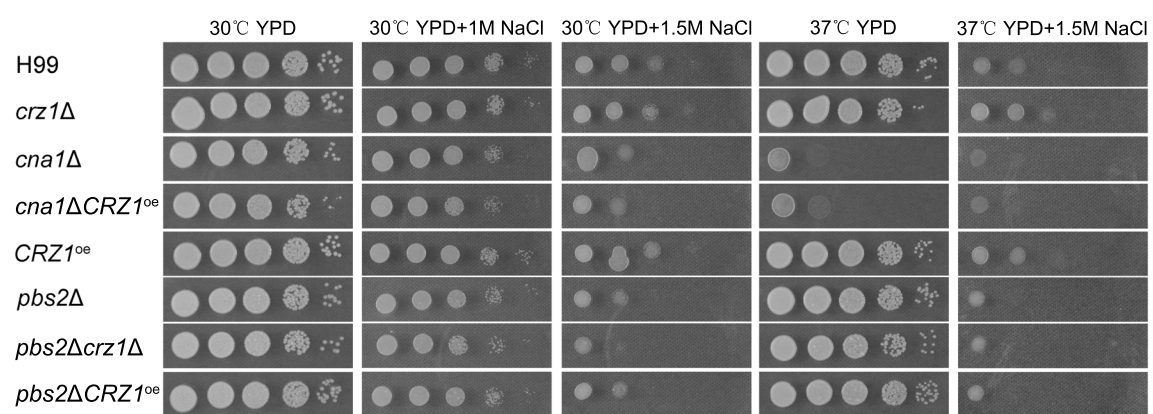

Supplement: S8 Fig — Cells of the following strains (wild-type H99, crz1Δ, cna1Δ, cna1ΔCRZ1oe, CRZ1oe, pbs2Δ, pbs2Δcrz1Δ, and pbs2ΔCRZ1oe) were serial diluted and spotted onto YPD medium with or without the addition of NaCl at 1M, or 1.5M. Cells were then incubated at 30°C or 37°C for 3 days before images were taken. (PDF) [file pgen.1006982.s008.pdf]
