# Supplementary material for: Glucosamine stimulates pheromone-independent dimorphic transition in Cryptococcus neoformans by promoting Crz1 nuclear translocation
Source: PLoS Genet. 2017 Sep 12;13(9):e1006982. doi: 10.1371/journal.pgen.1006982 (PMC5595294; doi:10.1371/journal.pgen.1006982)
Supplement: S1 Table — (DOCX) [file pgen.1006982.s009.docx]

Table 1-Forward Genetic Screening

| Screening result from Dr. Madhani’s deletion set: | |
| --- | --- |
| Non-Hyphal | |
| CNAG_00032 | 2,4-dihydroxyhept-2-ene-1,7-dioic acid aldolase |
| CNAG_00035 | hypothetical protein |
| CNAG_00039 | hypothetical protein |
| CNAG_00045 | hypothetical protein |
| CNAG_00156 | zinc finger transcription factor (SP1) |
| CNAG_00171 | peroxin-2 |
| CNAG_00187 | ubiquitin carboxyl-terminal hydrolase (UBP16) |
| CNAG_00289 | hypothetical protein |
| CNAG_00328 | DNA excision repair protein ERCC-5 |
| CNAG_00333 | hypothetical protein |
| CNAG_00399 | transformer-2-beta isoform 3 |
| CNAG_00413 | putative oxidoreductase (OFD1) |
| CNAG_00414 | hypothetical protein |
| CNAG_00422 | hypothetical protein |
| CNAG_00456 | hypothetical protein |
| CNAG_00498 | cell division cycle protein 14 |
| CNAG_00662 | carboxymethylenebutenolidase |
| CNAG_00698 | hypothetical protein |
| CNAG_00745 | serine/threonine-protein kinase (HRK1) |
| CNAG_01043 | hypothetical protein |
| CNAG_01230 | chitin-deacetylase (MP98) |
| CNAG_01243 | histone-lysine N-methyltransferase (SET101) |
| CNAG_01417 | hypothetical protein |
| CNAG_01452 | mat3 pheromone repeat protein (MAT3) |
| CNAG_01610 | hypothetical protein |
| CNAG_01718 | hypothetical protein |
| CNAG_01745 | glycerol-3-phosphate dehydrogenase (NAD(+)) |
| CNAG_01930 | endopeptidase |
| CNAG_01969 | zinc metalloprotease |
| CNAG_01970 | hypothetical protein |
| CNAG_02007 | adenylate kinase 1 |
| CNAG_02046 | poly(A)-binding protein binding protein |
| CNAG_02083 | siderochrome-iron transporter (SIT2) |
| CNAG_02141 | hypothetical protein |
| CNAG_02181 | dihydrokaempferol 4-reductase |
| CNAG_02221 | zinc ion transporter (ZIP3) |
| CNAG_02223 | signal sequence binding protein |
| CNAG_02473 | endoplasmic reticulum protein |
| CNAG_02551 | dihydroxyacetone kinase 1 |
| CNAG_02674 | hypothetical protein |
| CNAG_02703 | hypothetical protein |
| CNAG_02730 | sorting nexin-41 |
| CNAG_02736 | T-complex protein 1 subunit theta |
| CNAG_02776 | hypothetical protein |
| CNAG_03019 | long-chain acyl-CoA synthetase |
| CNAG_03079 | ER protein for association of GPI-anchored proteins with lipid rafts (PER1) |
| CNAG_03080 | fatty acid elongase |
| CNAG_03090 | hypothetical protein |
| CNAG_03139 | phosphatidylethanolamine N-methyltransferase (CHO2) |
| CNAG_03313 | hypothetical protein |
| CNAG_03316 | rho gdp-dissociation inhibitor (RDI1) |
| CNAG_03365 | hypothetical protein |
| CNAG_03406 | putative compass/set1c complex subunit (SPP101) |
| CNAG_03451 | hypothetical protein |
| CNAG_03452 | AFG1 family mitochondrial ATPase |
| CNAG_03590 | casein kinase II beta chain (CKB1) |
| CNAG_03664 | putative nickel transporter (NIC1) |
| CNAG_03928 | adaptor protein complex AP-1 |
| CNAG_04152 | protein phosphatase methylesterase 1 |
| CNAG_04224 | carboxy-terminal domain RNA polymerase II polypeptide A small phosphatase |
| CNAG_04373 | alginate lyase |
| CNAG_04408 | choline kinase |
| CNAG_04760 | cytoplasmic protein |
| CNAG_04804 | sterol regulatory element-binding protein (SRE1) |
| CNAG_04808 | XPG N-terminal domain-containing protein |
| CNAG_04842 | hypothetical protein |
| CNAG_04848 | mRNA binding protein |
| CNAG_05216 | CAMK protein kinase |
| CNAG_05253 | hypothetical protein |
| CNAG_05423 | SET domain-containing protein |
| CNAG_05533 | hypothetical protein |
| CNAG_05550 | hypothetical protein |
| CNAG_05667 | myo-inositol transporter (ITR3B) |
| CNAG_05675 | trimethyllysine dioxygenase |
| CNAG_05691 | hypothetical protein |
| CNAG_05715 | charged multivesicular body protein 5 |
| CNAG_05744 | 2-dehydropantoate 2-reductase |
| CNAG_05872 | endopeptidase |
| CNAG_05914 | MFS transporter, SP family, general alpha glucoside:H+ symporter |
| CNAG_06144 | cytoplasmic protein |
| CNAG_06193 | protein kinase (CRK1) |
| CNAG_06371 | guanine deaminase |
| CNAG_06383 | cytoplasmic protein |
| CNAG_06552 | CAMK/CAMKL/AMPK protein kinase (SNF1-related kinase complex anchoring protein SIP1?) |
| CNAG_06568 | RAN protein kinase |
| CNAG_06716 | hypothetical protein |
| CNAG_06863 | hypothetical protein |
| CNAG_06904 | hypothetical protein |
| CNAG_06925 | arsenical-resistance protein |
| CNAG_06968 | hypothetical protein |
| CNAG_07005 | armadillo/beta-catenin repeat protein; encoded on mat locus (BSP2) |
| CNAG_07311 | Set1/Ash2 histone methyltransferase complex subunit (BRE2) |
| CNAG_07329 | hypothetical protein |
| CNAG_07380 | hypothetical protein |
| CNAG_07410 | cid1-family polyA polymerase (CID1alpha) |
| CNAG_07567 | hypothetical protein |
| CNAG_07633 | hypothetical protein |
| CNAG_07670 | hypothetical protein |
| CNAG_00467 | hypothetical protein |
| CNAG_00564 | hypothetical protein |
| CNAG_01322 | hypothetical protein |
| CNAG_01643 | hypothetical protein |
| CNAG_02435 | white-collar transcription factor, blue-light photoresponsive gene (BWC2) |
| CNAG_03322 | UDP-glucuronic acid decarboxylase (UXS1) |
| CNAG_04514 | mitogen-activated protein kinase (MPK1) |
| CNAG_06648 | mRNA polymerase-associated protein (RTF1) |
| CNAG_07636 | putative chitin synthase regulator (CSR2) |
| CNAG_06401 | hypothetical protein |
| CNAG_00736 | exocyst protein |
| Decreased |  |
| CNAG_00053 | hypothetical protein |
| CNAG_00054 | hypothetical protein |
| CNAG_00149 | NADH dehydrogenase (ubiquinone) 1 alpha subcomplex 4 |
| CNAG_00177 | hypothetical protein |
| CNAG_00280 | complement component 1 Q subcomponent-binding protein, mitochondrial |
| CNAG_00287 | cytoplasmic protein |
| CNAG_00358 | tRNA dimethylallyltransferase |
| CNAG_00374 | hypothetical protein |
| CNAG_00394 | diphthamide biosynthesis protein 3 |
| CNAG_00490 | acetyl-CoA acyltransferase |
| CNAG_00540 | pantothenate transporter |
| CNAG_00568 | hypothetical protein |
| CNAG_00637 | cystathionine beta-synthase |
| CNAG_00679 | hypothetical protein |
| CNAG_00744 | alpha 1,6-mannosyltransferase (OCH1) |
| CNAG_00889 | hypothetical protein |
| CNAG_00995 | hypothetical protein |
| CNAG_01084 | ubiquitin conjugating enzyme (UBC4) |
| CNAG_01154 | hypothetical protein |
| CNAG_01239 | chitin deacetylase (CDA3) |
| CNAG_01350 | putative ubiquitin ligase (RMD5) |
| CNAG_01717 | cell differentiation protein rcd1 |
| CNAG_01815 | hypothetical protein |
| CNAG_01856 | hypothetical protein |
| CNAG_02102 | hypothetical protein |
| CNAG_02103 | hypothetical protein |
| CNAG_02147 | cytochrome c peroxidase |
| CNAG_02167 | vacuolar protein sorting-associated protein 27 |
| CNAG_02284 | 2-oxoisovalerate dehydrogenase E1 component, alpha subunit |
| CNAG_02285 | nucleoside diphosphate kinase |
| CNAG_02371 | coiled-coil domain-containing protein 130 |
| CNAG_02553 | short-chain dehydrogenase |
| CNAG_02554 | sugar transporter |
| CNAG_02673 | NAD dependent epimerase/dehydratase |
| CNAG_02735 | hypothetical protein |
| CNAG_02800 | hypothetical protein |
| CNAG_02819 | vacuolar membrane protein |
| CNAG_03048 | gluconokinase |
| CNAG_03125 | hypothetical protein |
| CNAG_03155 | ENTH domain-containing protein |
| CNAG_03404 | hypothetical protein |
| CNAG_03405 | hypothetical protein |
| CNAG_03517 | NADH dehydrogenase |
| CNAG_03722 | RAN protein binding protein |
| CNAG_03743 | glutamine amidotransferase |
| CNAG_03827 | hypothetical protein |
| CNAG_03977 | hypothetical protein |
| CNAG_04064 | hypothetical protein |
| CNAG_04068 | large subunit ribosomal protein L28e |
| CNAG_04237 | hypothetical protein |
| CNAG_04285 | hypothetical protein |
| CNAG_04570 | hypothetical protein |
| CNAG_04627 | COP9 signalosome complex subunit 12 |
| CNAG_04650 | actin-like protein ARP6 |
| CNAG_04772 | hypothetical protein |
| CNAG_04825 | hypothetical protein |
| CNAG_04904 | clathrin heavy chain |
| CNAG_04945 | hypothetical protein |
| CNAG_05192 | hypothetical protein |
| CNAG_05236 | hypothetical protein |
| CNAG_05308 | beta-catenin-like protein 1 |
| CNAG_05397 | hypothetical protein |
| CNAG_05422 | virulence related protein of unknown function (LIV11) |
| CNAG_05570 | hypothetical protein |
| CNAG_05662 | myo-inositol transporter (ITR4) |
| CNAG_05745 | cytoplasmic protein |
| CNAG_05838 | rho GTPase activating protein (GAP) homolog (RGD1) |
| CNAG_05892 | phosphoglycerate mutase |
| CNAG_06099 | hypothetical protein |
| CNAG_06141 | dUTP pyrophosphatase |
| CNAG_06294 | hypothetical protein |
| CNAG_06440 | DNA dependent ATPase |
| CNAG_06485 | hexose transporter protein |
| CNAG_06583 | hypothetical protein |
| CNAG_06649 | haloacid dehalogenase, type II |
| CNAG_06656 | diphthine synthase |
| CNAG_06777 | fructosyl amino acid oxidase |
| CNAG_07335 | cystinosin |
| CNAG_07479 | hypothetical protein |
| CNAG_07566 | hypothetical protein |
| CNAG_07608 | hypothetical protein |
| CNAG_07609 | ATP-dependent RNA helicase DDX51/DBP6 |
| CNAG_07677 | hypothetical protein |
| CNAG_07866 | transcription initiation factor TFIIA small subunit |
| CNAG_07903 | rho guanine nucleotide exchange factor (ROM20) |
| Increased |  |
| CNAG_00125 | regulator of G-protein signaling (CRG1) |
| CNAG_00404 | RING zinc finger protein |
| CNAG_00405 | ste/ste20/ysk protein kinase (KIC1) |
| CNAG_00436 | palmitoyltransferase AKR1 |
| CNAG_00484 | 2-oxoisovalerate dehydrogenase E2 component (dihydrolipoyl transacylase) |
| CNAG_00600 | capsule-associated protein (CAP60) |
| CNAG_00890 | hypothetical protein |
| CNAG_01126 | guanine nucleotide exchange protein for ADP-robosylation factor |
| CNAG_01523 | mitogen-activated protein kinase (HOG1) |
| CNAG_01688 | ATP-dependent metalloprotease |
| CNAG_02090 | heterotrimeric G-protein coupled receptor alpha subunit (GPA3) |
| CNAG_02270 | homoserine O-acetyltransferase (MET2) |
| CNAG_02702 | putative voltage-gated chloride channel (CLC1) |
| CNAG_02830 | C-24(28) sterol reductase (ERG4) |
| CNAG_02883 | ras-like gtp-binding protein (RAC1) |
| CNAG_02885 | capsule-associated protein (CAP64) |
| CNAG_03324 | hypothetical protein |
| CNAG_03385 | cyclin (PCL103) |
| CNAG_03449 | DNA repair protein |
| CNAG_03858 | hypothetical protein |
| CNAG_04649 | peptide alpha-N-acetyltransferase |
| CNAG_05147 | hypothetical protein |
| CNAG_05583 | ceramide glucosyltransferase (GCS1) |
| CNAG_05695 | glucosamine 6-phosphate N-acetyltransferase |
| CNAG_05842 | cytochrome P450 |
| CNAG_06298 | hypothetical protein |
| CNAG_06469 | aminophospholipid translocase(flippase) (APT1) |
| CNAG_07320 | hypothetical protein |
| CNAG_07401 | hypothetical protein |
| CNAG_07415 | hypothetical protein |
| CNAG_07699 | zinc finger family protein |
| CNAG_00266 | hypothetical protein |
| CNAG_00293 | G-protein signaling GTPase (RAS1) |
| CNAG_00391 | hypothetical protein |
| CNAG_00495 | hypothetical protein |
| CNAG_00772 | putative UV excision repair protein (RAD23) |
| CNAG_00773 | hypothetical protein |
| CNAG_01005 | glutaredoxin |
| CNAG_01029 | impact family protein |
| CNAG_01324 | hypothetical protein |
| CNAG_01401 | hypothetical protein |
| CNAG_01418 | hypothetical protein |
| CNAG_01489 | maltose O-acetyltransferase (CAS9) |
| CNAG_01525 | hypothetical protein |
| CNAG_01723 | hypothetical protein |
| CNAG_01735 | hypothetical protein |
| CNAG_01816 | hypothetical protein |
| CNAG_02148 | ubiquitin-conjugating enzyme E2 35 |
| CNAG_02224 | hypothetical protein |
| CNAG_02349 | hypothetical protein |
| CNAG_02771 | DNA repair and recombination protein RAD54B |
| CNAG_02845 | hypothetical protein |
| CNAG_03039 | DNA damage-inducible protein 1 |
| CNAG_03203 | DNA polymerase epsilon p12 subunit |
| CNAG_03206 | endonuclease III |
| CNAG_03403 | mitochondrial protein |
| CNAG_03500 | osmosensor |
| CNAG_03741 | hypothetical protein |
| CNAG_04238 | peroxisomal 2,4-dienoyl-CoA reductase |
| CNAG_04272 | CAMK/CAMK1 protein kinase |
| CNAG_04417 | alpha-ketoglutarate-dependent 2,4-dichlorophenoxyacetate dioxygenase |
| CNAG_04450 | chromodomain-helicase-DNA-binding protein 1 |
| CNAG_04576 | hypothetical protein |
| CNAG_04639 | hypothetical protein |
| CNAG_04955 | oxidoreductase |
| CNAG_04956 | high-affinity nicotinic acid transporter |
| CNAG_05003 | hypothetical protein |
| CNAG_05134 | ornithine-oxo-acid transaminase |
| CNAG_05377 | myo-inositol transporter (ITR3) |
| CNAG_05402 | protein CMS1 |
| CNAG_05508 | hypothetical protein |
| CNAG_05724 | hypothetical protein |
| CNAG_05913 | alpha-glucosidase |
| CNAG_06145 | hypothetical protein |
| CNAG_06295 | cytoplasmic protein |
| CNAG_06313 | phosphoglucomutase |
| CNAG_06332 | hypothetical protein |
| CNAG_06402 | 26S proteasome complex subunit DSS1 |
| CNAG_06403 | hypothetical protein |
| CNAG_06484 | hypothetical protein |
| CNAG_06772 | nucleoporin SEH1 |
| CNAG_07319 | hypothetical protein |
| CNAG_07523 | hypothetical protein |
| Hyperfilamentation |  |
| CNAG_00003 | drug transporter |
| CNAG_00531 | p-type ATPase sodium pump (ENA1) |
| CNAG_01850 | two-component-like sensor kinase (TCO1) |
| CNAG_01855 | hypothetical protein |
| CNAG_01891 | RAD57 protein |
| CNAG_02179 | hemolysin |
| CNAG_02393 | hypothetical protein |
| CNAG_02676 | hypothetical protein |
| CNAG_02930 | virulence related protein of unknown function (LIV10) |
| CNAG_03234 | cell wall surface anchor protein |
| CNAG_03504 | hypothetical protein |
| CNAG_04565 | hypothetical protein |
| CNAG_05176 | hypothetical protein |
| CNAG_05195 | ubiquitin-conjugation factor E4 B |
| CNAG_05420 | RNA polymerase II transcription factor |
| CNAG_05940 | hypothetical protein |
| CNAG_06555 | aromatic amino acid aminotransferase I |
| CNAG_06680 | COP9 signalosome complex subunit 6 |
| CNAG_06698 | phosphatase |
| CNAG_06401 | hypothetical protein |
| CNAG_00736 | exocyst protein |
| Growth defect |  |
| CNAG_00051 | putative set3c deacetylase complex subunit (SNT1) |
| CNAG_01360 | pumilio domain-containing protein c |
| CNAG_02122 | cytoplasmic tRNA 2-thiolation protein 1 |
| CNAG_02795 | phosphoribosylglycinamide formyltransferase |
| CNAG_02891 | endoplasmic reticulum protein |
| CNAG_03431 | nuclear protein |
| CNAG_03482 | thiol peroxidase (TSA1) |
| CNAG_03940 | elongator complex protein 2 |
| CNAG_03965 | ribosome recycling factor |
| CNAG_04186 | hypothetical protein |
| CNAG_04570 | hypothetical protein |
| CNAG_04992 | hypothetical protein |
| CNAG_06191 | hypothetical protein |
| CNAG_06383 | cytoplasmic protein |
| CNAG_06761 | siderophore-iron transporter Str1 |
| CNAG_07311 | Set1/Ash2 histone methyltransferase complex subunit (BRE2) |
| CNAG_07317 | hypothetical protein |
| CNAG_07373 | carbamoyl-phosphate synthase, large subunit |
| Screening result from Dr. Bahn’s TFs deletion set | |
| Non-Hyphal | |
| CNAG_00156 | SP1 |
| CNAG_00559 | BZP3 |
| CNAG_03366 | ZNF2 |
| CNAG_05019 | HOB6 |
| CNAG_07059 | FAP1 |
| CNAG_03561 | FZC33 |
| CNAG_04263 | BZP2 |
| Decreased |  |
| CNAG_04583 | DDT1 |
| Increased |  |
| CNAG_00514 | GAT6-ALSO YSB1385 |
| CNAG_04353 | CLR1 |
| CNAG_04586 | HOB7 |
| CNAG_04908 | CLR4 |
| Hyperfilamentation |  |
| CNAG_02566 | FKH2 |
| CNAG_07924 | MCM1 |
| Growth defect |  |
| CNAG_04804 | SRE1 |
| CNAG_01431 | HOB1 |
| CNAG_01626 | ADA2 |
| CNAG_06188 | FZC15 |
