# Supplementary material for: Glucosamine stimulates pheromone-independent dimorphic transition in Cryptococcus neoformans by promoting Crz1 nuclear translocation
Source: PLoS Genet. 2017 Sep 12;13(9):e1006982. doi: 10.1371/journal.pgen.1006982 (PMC5595294; doi:10.1371/journal.pgen.1006982)
Supplement: S2 Table — (DOCX) [file pgen.1006982.s010.docx]

**Supplemental Table 2. Strains Used in This Study.**

| **Strains** | **Genotype** | **Source** |
| --- | --- | --- |
| deletion set | 2000 mutants deleted individually by Dr. Madani’s Group, 2015 | FGSC |
| TF deletion set | all putative transcription factors genes deleted by Bahn’s Group | Bahn YS[[1](#_ENREF_1)] |
| Kinase deletion set | all putative kinase genes deleted by Bahn’s Group | Bahn YS[[2](#_ENREF_2)] |
| KR333-4 | VGI, serotype B, Tree hollow, South Africa | Perfect J[[3](#_ENREF_3)] |
| KR336-6 | VGI, serotype B, Tree hollow, South Africa | Perfect J[[3](#_ENREF_3)] |
| KR336-7 | VGI, serotype B, Tree hollow, South Africa | Perfect J[[3](#_ENREF_3)] |
| D17-4 | VNI-A2/M2 Pigeon excreta, South Africa | Perfect J[[3](#_ENREF_3)] |
| 7375 | VGII, clinical isolate, USA | Perfect J[[3](#_ENREF_3)] |
| 7729 | VGI, clinical isolate | Perfect J[[3](#_ENREF_3)] |
| 7730 | VGI, clinical isolate | Perfect J[[3](#_ENREF_3)] |
| 7731 | VGI, clinical isolate | Perfect J[[3](#_ENREF_3)] |
| 7734 | VGIIa, clinical isolate, USA | Perfect J[[3](#_ENREF_3)] |
| 7745 | VGIII, clinical isolate, USA | Perfect J[[3](#_ENREF_3)] |
| 35-20 | VNI, Pigeon excreta, USA | Perfect J[[3](#_ENREF_3)] |
| 35-21 | VNIII, AD hybrid, Pigeon excreta, USA | Perfect J[[3](#_ENREF_3)] |
| 84-1 | VNI-A4/M4, Pigeon excreta, USA | Perfect J[[3](#_ENREF_3)] |
| 84-12 | VNI, Pigeon excreta, USA | Perfect J[[3](#_ENREF_3)] |
| 8052 | VGIIa, vet isolate, USA | Perfect J[[3](#_ENREF_3)] |
| 8370 | VGII, clinical isolate, Brazil | Perfect J[[3](#_ENREF_3)] |
| 8146 | VGIIc, vet isolate, USA | Perfect J[[3](#_ENREF_3)] |
| 8106 | VGIIc, vet isolate, USA | Perfect J[[3](#_ENREF_3)] |
| 8105 | VGIIa, vet isolate, USA | Perfect J[[3](#_ENREF_3)] |
| 8104 | VGIIb, vet isolate, USA | Perfect J[[3](#_ENREF_3)] |
| 8103 | VGIIc, vet isolate, USA | Perfect J[[3](#_ENREF_3)] |
| 8102 | VGIIa, vet isolate, USA | Perfect J[[3](#_ENREF_3)] |
| 84-20 | VNI, Pigeon excreta, USA | Perfect J[[3](#_ENREF_3)] |
| KK1 JH#5826 | *cna1*::NAT, H99 | Heitman J[[4-6](#_ENREF_4)] |
| KK2 JH#5827 | *cnb1*::NAT, H99 | Heitman J[[4-6](#_ENREF_4)] |
| KK8 JH#5833 | *cna1*::NEO, KN99 | Heitman J[[4-6](#_ENREF_4)] |
| KK9 JH#5834 | *cna1*::NEO, KN99 | Heitman J[[4-6](#_ENREF_4)] |
| KK15 JH#5840 | *cnb1*::NAT, KN99 | Heitman J[[4-6](#_ENREF_4)] |
| KK19 JH#5844 | *cbp1*::NAT, H99 | Heitman J[[4-6](#_ENREF_4)] |
| KK22 JH#5847 | *cbp1*::NEO, KN99 | Heitman J[[4-6](#_ENREF_4)] |
| KK33 JH#5858 | *pcl1*::NAT, KN99 | Heitman J[[4-6](#_ENREF_4)] |
| YSB278 | MATalpha *tco1*::NAT | Bahn YS[[7](#_ENREF_7)] |
| YSB355 | MATalpha, *tco1*::NATSTM#102 + *TCO1*-NEO | Bahn YS[[7](#_ENREF_7)] |
| YSB281 | MATalpha, *tco2*::NAT | Bahn YS[[7](#_ENREF_7)] |
| 5A7 | MATalpha, *CNAG_00156* (*crz1*)::NAT | FGSC |
| TF1E12 | MATalpha, *CNAG_00156* (*crz1*)::NAT | Bahn YS[1] |
| TF1F12 | MATalpha, *CNAG_00156* (*crz1*)::NAT | Bahn YS[1] |
| XX266 | MATalpha, P*_GPD1_*- mCherry*-CRZ1* (NEO) | This study |
| XX429 | MATalpha, P*_GPD1_*- mCherry*-CRZ1* (NEO) | This study |
| XX453 | MATalpha, P*_GPD_*_1_- mCherry-*CRZ*1 (NEO), *cnb1*::NAT | This study |
| XX479 | MATalpha, P*_GPD1_*-*ZNF2*-AsisI V5 (NEO), *crz1*::NAT | This study |
| XX505 | MAT**a**, P*_GPD1_*-mCherry-AsisI-*CRZ1* (NEO) | This study |
| XX507 | MAT**a**, P*_GPD1_*-mCherry-AsisI-*CRZ1* (NEO) | This study |
| JL410 | MATalpha, P*_GPD1_*-mCherry-AsisI-*CRZ1* (NEO) | This study |
| XX509 | MATalpha, *crz1*::NAT, P*_GPD1_*-mCherry-AsisI-*CRZ1* (NEO) | This study |
| XX510 | MATalpha, *crz1*::NAT, P*_GPD1_*-mCherry-AsisI-*CRZ1* (NEO) | This study |
| XX513 | MATalpha, *znf2*::NAT, P*_GPD1_*-mCherry-AsisI-*CRZ1* (NEO) | This study |
| XX516 | MATalpha, *znf2*::NAT, P*_GPD1_*-mCherry-AsisI-*CRZ1* (NEO) | This study |
| XX10 | P*_CTR4_*-mCherry-*ZNF2* (NEO) | This study |
| XX522 | MATalpha, *crz1*::NAT, P*_CTR4_*-mCherry-AsisI-*ZNF2* (NEO) | This study |
| XX524 | MATalpha, *crz1*::NAT, P*_CTR4_*-mCherry-AsisI-*ZNF2* (NEO) | This study |
| YSB261 | MATalpha, *CNAG_05063*(s*sk1*)::NAT | Bahn YS[[7](#_ENREF_7)] |
| YSB428 | MATalpha, *ssk1*::NATSTM#205, *SSK1*-NEO | Bahn YS[[7](#_ENREF_7)] |
| XX531 | MATalpha, *CNAG_05063*(s*sk2*)::NAT | This study |
| YSB367 | MATalpha, *ssk2*::NATSTM#210, SSK2-NEO | Bahn YS[[7](#_ENREF_7)] |
| XX534 | MATalpha, *CNAG_00769* (*pbs2*)::NAT | Bahn YS[[7](#_ENREF_7)] |
| YSB212 | MATalpha, *pbs2*::NATSTM#123, *PBS2*-NEO | Bahn YS[[7](#_ENREF_7)] |
| Linlab4670 | MAT**a**, *hog1*::NAT | This study |
| YSB64 | MATalpha, *hog1*::NAT | Bahn YS[[7](#_ENREF_7)] |
| JL412 | MATalpha, P*_GPD1_*-mCherry-AsisI-CRZ1 (NEO) ; *CNAG_00769* (*pbs2*)::NAT | This study |
| XX543 | MAT**a**, P*_GDP1_*-mCherry-AsisI-CRZ1 (NEO), *CNAG_05063* (*ssk2*)::NAT | This study |
| XX545 | MAT**a**, P*_GDP1_*-mCherry-AsisI-CRZ1 (NEO), *CNAG_05063* (*ssk2*)::NAT | This study |
| XX546 | MATalpha, P*_GPD1_*-mCherry-AsisI-CRZ1 (NEO), *CNAG_00769* (*pbs2*)::NAT | This study |
| JL413 | MAT**a**, *crz1*::NAT*; pbs2*::NAT | This study |
| JL414 | MATalpha, *crz1*::NAT; *pbs2*::NAT | This study |
| JL415 | MAT**a**, P*_GPD1_*-mCherry-AsisI-*CRZ1* (NEO); *pbs2*::NAT | This study |
| JL416 | MAT**a**, P*_GPD1_*-mCherry-AsisI-*CRZ1* (NEO); *CNAG_05063*(*ssk2*)::NAT | This study |
| JL407 | MAT**a**, P*_GPD1_*-mCherry-AsisI-*CRZ1* (NEO); *hog1*::NAT | This study |
| JL408 | MATalpha, P*_GPD1_*-mCherry-AsisI-*CRZ1* (NEO); *hog1*::NAT | This study |
| JL322 | MAT**a**, P*_CRZ1_*-*CRZ1*-mCherry(NEO); GFP-NOP1::NAT; *ssk1*::NAT | This study |
| JL330 | MAT**a**, P*_CRZ1_*-*CRZ1*-mCherry(NEO); GFP-NOP1::NAT; *ssk2*::NAT | This study |
| JL338 | MAT**a**, P*_CRZ1_*-*CRZ1*-mCherry(NEO); GFP-NOP1::NAT; *pbs2*::NAT | This study |
| JL342 | MATalpha, *cna1*::NAT, P*_GPD1_*-mCherry-*CRZ1*(NEO) | This study |
| JL344 | MATalpha, *cnb1*::NAT, P*_GPD1_*-mCherry-*CRZ1*(NEO) | This study |
| JL348 | MATalpha, *cbp1*::NAT, P*_GPD1_*-mCherry-*CRZ1*(NEO) | This study |
| YPH71 | MATalpha *ste3*::NEO | Hseuh YP[8] |
| YPH139 | ste3a +STE3a-DsRED #5 | Hseuh YP[8] |
| YPH16 | MATalpha *cpr2*::NAT | Hseuh YP[[8](#_ENREF_8)] |
| YSB83 | MATalpha *gpa1*::NATSTM#5 | Bahn YS[[9](#_ENREF_9)] |
| YSB49 | MATalpha *gpb1*::NAT | Bahn YS[9] |
| JF219 | MATalpha *ste20a*::NEO *ura5* | Wang P[[10](#_ENREF_10)] |
| YSB345 | MATalpha, *ste7*::NEO | Bahn YS[9] |
| JL123 | MATalpha, *cpk1*::NAT | This study |
| XL1601 | MATalpha, *znf2*::NEO | Lin X[[11](#_ENREF_11)] |
| H99α | wild type | Nielsen K[[12](#_ENREF_12)] |
| H99**a** | wild type | Nielsen K[12] |
| XL280α | wild type | Lin X[[13](#_ENREF_13)] |
| XL280a | wild type | Zhai B[[14](#_ENREF_14)] |
| YSB342 | H99**a**, *ste7*::NATSTM#225 | Bahn YS[9] |
| YSB343 | H99**a**, *ste7*::NATSTM#225 | Bahn YS[9] |
| YSB344 | H99**a**, *ste7*::NATSTM#225 | Bahn YS[9] |
| YSB346 | MATalpha, *ste7*::NEO | Bahn YS[9] |
| YSB347 | MATalpha, *ste7*::NEO | Bahn YS[9] |
| *C. albicans* SC5314 | WT strain, Ura+ parent of CAI4 | NRCA [15] |
| *C. albicans* 3320 | (alpha/alpha) trp1/trp1 lys2/lys2 | NRCA |
| *C. albicans* 3316 | (**a**/**a**) arg/arg ilv/ilv | NRCA |

FGSC: Fungal Genetics Stock Center

1. Jung KW, Yang DH, Maeng S, Lee KT, So YS, Hong J, et al. Systematic functional profiling of transcription factor networks in *Cryptococcus neoformans*. Nature communications. 2015;6:6757. doi: 10.1038/ncomms7757. PubMed PMID: 25849373; PubMed Central PMCID: PMCPMC4391232.

2. Lee K-T, So Y-S, Yang D-H, Jung K-W, Choi J, Lee D-G, et al. Systematic functional analysis of kinases in the fungal pathogen Cryptococcus neoformans. Nat Commun. 2016;7. doi: 10.1038/ncomms12766.

3. Frazzitta AE, Vora H, Price MS, Tenor JL, Betancourt-Quiroz M, Toffaletti DL, et al. Nitrogen source-dependent capsule induction in human-pathogenic *Cryptococcus* species. Eukaryotic cell. 2013;12(11):1439-50. doi: 10.1128/EC.00169-13. PubMed PMID: 23975889; PubMed Central PMCID: PMC3837930.

4. Cruz MC, Fox DS, Heitman J. Calcineurin is required for hyphal elongation during mating and haploid fruiting in *Cryptococcus neoformans*. The EMBO journal. 2001;20(5):1020-32. PubMed PMID: 11230126.

5. Gorlach J, Fox DS, Cutler NS, Cox GM, Perfect JR, Heitman J. Identification and characterization of a highly conserved calcineurin binding protein, CBP1/calcipressin, in Cryptococcus neoformans. The EMBO journal. 2000;19(14):3618-29. doi: 10.1093/emboj/19.14.3618. PubMed PMID: 10899116; PubMed Central PMCID: PMCPMC313974.

6. Odom A, Muir S, Lim E, Toffaletti DL, Perfect J, Heitman J. Calcineurin is required for virulence of Cryptococcus neoformans. The EMBO journal. 1997;16(10):2576-89. doi: 10.1093/emboj/16.10.2576.

7. Bahn YS, Kojima K, Cox GM, Heitman J. A unique fungal two-component system regulates stress responses, drug sensitivity, sexual development, and virulence of *Cryptococcus neoformans*. Molecular biology of the cell. 2006;17(7):3122-35. PubMed PMID: 16672377.

8. Hsueh YP, Xue C, Heitman J. A constitutively active GPCR governs morphogenic transitions in *Cryptococcus neoformans*. The EMBO journal. 2009;28(9):1220-33. PubMed PMID: 19322200.

9. Bahn YS, Hicks JK, Giles SS, Cox GM, Heitman J. Adenylyl cyclase-associated protein Aca1 regulates virulence and differentiation of *Cryptococcus neoformans* via the cyclic AMP-protein kinase A cascade. Eukaryotic cell. 2004;3(6):1476-91. PubMed PMID: 15590822.

10. Wang P, Nichols CB, Lengeler KB, Cardenas ME, Cox GM, Perfect JR, et al. Mating-type-specific and nonspecific PAK kinases play shared and divergent roles in *Cryptococcus neoformans*. Eukaryotic cell. 2002;1(2):257-72. PubMed PMID: 12455960.

11. Lin X, Jackson JC, Feretzaki M, Xue C, Heitman J. Transcription factors Mat2 and Znf2 operate cellular circuits orchestrating opposite- and same-sex mating in *Cryptococcus neoformans*. PLoS genetics. 2010;6(5):e1000953. Epub 2010/05/21. doi: 10.1371/journal.pgen.1000953. PubMed PMID: 20485569; PubMed Central PMCID: PMC2869318.

12. Nielsen K, Cox GM, Wang P, Toffaletti DL, Perfect JR, Heitman J. Sexual cycle of *Cryptococcus neoformans* var. *grubii* and virulence of congenic **a** and alpha isolates. Infection and immunity. 2003;71(9):4831-41. PubMed PMID: 12933823.

13. Lin X, Hull CM, Heitman J. Sexual reproduction between partners of the same mating type in *Cryptococcus neoformans*. Nature. 2005;434(7036):1017-21. PubMed PMID: 15846346.

14. Zhai B, Zhu P, Foyle D, Upadhyay S, Idnurm A, Lin X. Congenic strains of the filamentous form of *Cryptococcus neoformans* for studies of fungal morphogenesis and virulence. Infection and immunity. 2013;81(7):2626-37. Epub 2013/05/15. doi: 10.1128/IAI.00259-13. PubMed PMID: 23670559; PubMed Central PMCID: PMC3697605.

1. Jung KW, Yang DH, Maeng S, Lee KT, So YS, Hong J, et al. Systematic functional profiling of transcription factor networks in *Cryptococcus neoformans*. Nature communications. 2015;6:6757. doi: 10.1038/ncomms7757. PubMed PMID: 25849373; PubMed Central PMCID: PMCPMC4391232.

2. Lee K-T, So Y-S, Yang D-H, Jung K-W, Choi J, Lee D-G, et al. Systematic functional analysis of kinases in the fungal pathogen Cryptococcus neoformans. Nat Commun. 2016;7. doi: 10.1038/ncomms12766.

3. Frazzitta AE, Vora H, Price MS, Tenor JL, Betancourt-Quiroz M, Toffaletti DL, et al. Nitrogen source-dependent capsule induction in human-pathogenic *Cryptococcus* species. Eukaryotic cell. 2013;12(11):1439-50. doi: 10.1128/EC.00169-13. PubMed PMID: 23975889; PubMed Central PMCID: PMC3837930.

4. Cruz MC, Fox DS, Heitman J. Calcineurin is required for hyphal elongation during mating and haploid fruiting in *Cryptococcus neoformans*. The EMBO journal. 2001;20(5):1020-32. PubMed PMID: 11230126.

5. Gorlach J, Fox DS, Cutler NS, Cox GM, Perfect JR, Heitman J. Identification and characterization of a highly conserved calcineurin binding protein, CBP1/calcipressin, in Cryptococcus neoformans. The EMBO journal. 2000;19(14):3618-29. doi: 10.1093/emboj/19.14.3618. PubMed PMID: 10899116; PubMed Central PMCID: PMCPMC313974.

6. Odom A, Muir S, Lim E, Toffaletti DL, Perfect J, Heitman J. Calcineurin is required for virulence of Cryptococcus neoformans. The EMBO journal. 1997;16(10):2576-89. doi: 10.1093/emboj/16.10.2576.

7. Bahn YS, Kojima K, Cox GM, Heitman J. A unique fungal two-component system regulates stress responses, drug sensitivity, sexual development, and virulence of *Cryptococcus neoformans*. Molecular biology of the cell. 2006;17(7):3122-35. PubMed PMID: 16672377.

8. Hsueh YP, Xue C, Heitman J. A constitutively active GPCR governs morphogenic transitions in *Cryptococcus neoformans*. The EMBO journal. 2009;28(9):1220-33. PubMed PMID: 19322200.

9. Bahn YS, Hicks JK, Giles SS, Cox GM, Heitman J. Adenylyl cyclase-associated protein Aca1 regulates virulence and differentiation of *Cryptococcus neoformans* via the cyclic AMP-protein kinase A cascade. Eukaryotic cell. 2004;3(6):1476-91. PubMed PMID: 15590822.

10. Wang P, Nichols CB, Lengeler KB, Cardenas ME, Cox GM, Perfect JR, et al. Mating-type-specific and nonspecific PAK kinases play shared and divergent roles in *Cryptococcus neoformans*. Eukaryotic cell. 2002;1(2):257-72. PubMed PMID: 12455960.

11. Lin X, Jackson JC, Feretzaki M, Xue C, Heitman J. Transcription factors Mat2 and Znf2 operate cellular circuits orchestrating opposite- and same-sex mating in *Cryptococcus neoformans*. PLoS genetics. 2010;6(5):e1000953. Epub 2010/05/21. doi: 10.1371/journal.pgen.1000953. PubMed PMID: 20485569; PubMed Central PMCID: PMC2869318.

12. Nielsen K, Cox GM, Wang P, Toffaletti DL, Perfect JR, Heitman J. Sexual cycle of *Cryptococcus neoformans* var. *grubii* and virulence of congenic **a** and alpha isolates. Infection and immunity. 2003;71(9):4831-41. PubMed PMID: 12933823.

13. Lin X, Hull CM, Heitman J. Sexual reproduction between partners of the same mating type in *Cryptococcus neoformans*. Nature. 2005;434(7036):1017-21. PubMed PMID: 15846346.

14. Zhai B, Zhu P, Foyle D, Upadhyay S, Idnurm A, Lin X. Congenic strains of the filamentous form of *Cryptococcus neoformans* for studies of fungal morphogenesis and virulence. Infection and immunity. 2013;81(7):2626-37. Epub 2013/05/15. doi: 10.1128/IAI.00259-13. PubMed PMID: 23670559; PubMed Central PMCID: PMC3697605.

1. Jung KW, Yang DH, Maeng S, Lee KT, So YS, Hong J, et al. Systematic functional profiling of transcription factor networks in *Cryptococcus neoformans*. Nature communications. 2015;6:6757. doi: 10.1038/ncomms7757. PubMed PMID: 25849373; PubMed Central PMCID: PMCPMC4391232.

2. Lee K-T, So Y-S, Yang D-H, Jung K-W, Choi J, Lee D-G, et al. Systematic functional analysis of kinases in the fungal pathogen Cryptococcus neoformans. Nat Commun. 2016;7. doi: 10.1038/ncomms12766.

3. Frazzitta AE, Vora H, Price MS, Tenor JL, Betancourt-Quiroz M, Toffaletti DL, et al. Nitrogen source-dependent capsule induction in human-pathogenic *Cryptococcus* species. Eukaryotic cell. 2013;12(11):1439-50. doi: 10.1128/EC.00169-13. PubMed PMID: 23975889; PubMed Central PMCID: PMC3837930.

4. Cruz MC, Fox DS, Heitman J. Calcineurin is required for hyphal elongation during mating and haploid fruiting in *Cryptococcus neoformans*. The EMBO journal. 2001;20(5):1020-32. PubMed PMID: 11230126.

5. Gorlach J, Fox DS, Cutler NS, Cox GM, Perfect JR, Heitman J. Identification and characterization of a highly conserved calcineurin binding protein, CBP1/calcipressin, in Cryptococcus neoformans. The EMBO journal. 2000;19(14):3618-29. doi: 10.1093/emboj/19.14.3618. PubMed PMID: 10899116; PubMed Central PMCID: PMCPMC313974.

6. Odom A, Muir S, Lim E, Toffaletti DL, Perfect J, Heitman J. Calcineurin is required for virulence of Cryptococcus neoformans. The EMBO journal. 1997;16(10):2576-89. doi: 10.1093/emboj/16.10.2576.

7. Bahn YS, Kojima K, Cox GM, Heitman J. A unique fungal two-component system regulates stress responses, drug sensitivity, sexual development, and virulence of *Cryptococcus neoformans*. Molecular biology of the cell. 2006;17(7):3122-35. PubMed PMID: 16672377.

8. Hsueh YP, Xue C, Heitman J. A constitutively active GPCR governs morphogenic transitions in *Cryptococcus neoformans*. The EMBO journal. 2009;28(9):1220-33. PubMed PMID: 19322200.

9. Bahn YS, Hicks JK, Giles SS, Cox GM, Heitman J. Adenylyl cyclase-associated protein Aca1 regulates virulence and differentiation of *Cryptococcus neoformans* via the cyclic AMP-protein kinase A cascade. Eukaryotic cell. 2004;3(6):1476-91. PubMed PMID: 15590822.

10. Wang P, Nichols CB, Lengeler KB, Cardenas ME, Cox GM, Perfect JR, et al. Mating-type-specific and nonspecific PAK kinases play shared and divergent roles in *Cryptococcus neoformans*. Eukaryotic cell. 2002;1(2):257-72. PubMed PMID: 12455960.

11. Lin X, Jackson JC, Feretzaki M, Xue C, Heitman J. Transcription factors Mat2 and Znf2 operate cellular circuits orchestrating opposite- and same-sex mating in *Cryptococcus neoformans*. PLoS genetics. 2010;6(5):e1000953. Epub 2010/05/21. doi: 10.1371/journal.pgen.1000953. PubMed PMID: 20485569; PubMed Central PMCID: PMC2869318.

12. Nielsen K, Cox GM, Wang P, Toffaletti DL, Perfect JR, Heitman J. Sexual cycle of *Cryptococcus neoformans* var. *grubii* and virulence of congenic **a** and alpha isolates. Infection and immunity. 2003;71(9):4831-41. PubMed PMID: 12933823.

13. Lin X, Hull CM, Heitman J. Sexual reproduction between partners of the same mating type in *Cryptococcus neoformans*. Nature. 2005;434(7036):1017-21. PubMed PMID: 15846346.

14. Zhai B, Zhu P, Foyle D, Upadhyay S, Idnurm A, Lin X. Congenic strains of the filamentous form of *Cryptococcus neoformans* for studies of fungal morphogenesis and virulence. Infection and immunity. 2013;81(7):2626-37. Epub 2013/05/15. doi: 10.1128/IAI.00259-13. PubMed PMID: 23670559; PubMed Central PMCID: PMC3697605.

1. Jung KW, Yang DH, Maeng S, Lee KT, So YS, Hong J, et al. Systematic functional profiling of transcription factor networks in *Cryptococcus neoformans*. Nat Commun. 2015;6:6757. doi: 10.1038/ncomms7757. PubMed PMID: 25849373.

2. Lee K-T, So Y-S, Yang D-H, Jung K-W, Choi J, Lee D-G, et al. Systematic functional analysis of kinases in the fungal pathogen Cryptococcus neoformans. Nat Commun. 2016;7. doi: 10.1038/ncomms12766.

3. Frazzitta AE, Vora H, Price MS, Tenor JL, Betancourt-Quiroz M, Toffaletti DL, et al. Nitrogen source-dependent capsule induction in human-pathogenic *cryptococcus* species. Eukaryotic cell. 2013;12(11):1439-50. Epub 2013/08/27. doi: 10.1128/ec.00169-13. PubMed PMID: 23975889; PubMed Central PMCID: PMCPmc3837930.

4. Cruz MC, Fox DS, Heitman J. Calcineurin is required for hyphal elongation during mating and haploid fruiting in Cryptococcus neoformans. The EMBO journal. 2001;20(5):1020-32. Epub 2001/03/07. doi: 10.1093/emboj/20.5.1020. PubMed PMID: 11230126; PubMed Central PMCID: PMCPmc145507.

5. Gorlach J, Fox DS, Cutler NS, Cox GM, Perfect JR, Heitman J. Identification and characterization of a highly conserved calcineurin binding protein, CBP1/calcipressin, in Cryptococcus neoformans. The EMBO journal. 2000;19(14):3618-29. Epub 2000/07/19. doi: 10.1093/emboj/19.14.3618. PubMed PMID: 10899116; PubMed Central PMCID: PMCPmc313974.

6. Odom A, Muir S, Lim E, Toffaletti DL, Perfect J, Heitman J. Calcineurin is required for virulence of Cryptococcus neoformans. The EMBO journal. 1997;16(10):2576-89. doi: 10.1093/emboj/16.10.2576.

7. Bahn YS, Kojima K, Cox GM, Heitman J. A unique fungal two-component system regulates stress responses, drug sensitivity, sexual development, and virulence of *Cryptococcus neoformans*. Mol Biol Cell. 2006;17(7):3122-35. Epub 2006/05/05. doi: 10.1091/mbc.E06-02-0113. PubMed PMID: 16672377; PubMed Central PMCID: PMCPmc1483045.

8. Hsueh YP, Xue C, Heitman J. A constitutively active GPCR governs morphogenic transitions in *Cryptococcus neoformans*. The EMBO journal. 2009;28(9):1220-33. Epub 2009/03/27. doi: 10.1038/emboj.2009.68. PubMed PMID: 19322200; PubMed Central PMCID: PMCPmc2683048.

9. Bahn YS, Hicks JK, Giles SS, Cox GM, Heitman J. Adenylyl cyclase-associated protein Aca1 regulates virulence and differentiation of Cryptococcus neoformans via the cyclic AMP-protein kinase A cascade. Eukaryotic cell. 2004;3(6):1476-91. Epub 2004/12/14. doi: 10.1128/ec.3.6.1476-1491.2004. PubMed PMID: 15590822; PubMed Central PMCID: PMCPmc539029.

10. Wang P, Nichols CB, Lengeler KB, Cardenas ME, Cox GM, Perfect JR, et al. Mating-type-specific and nonspecific PAK kinases play shared and divergent roles in Cryptococcus neoformans. Eukaryotic cell. 2002;1(2):257-72. Epub 2002/11/29. PubMed PMID: 12455960; PubMed Central PMCID: PMCPmc118036.

11. Lin X, Jackson JC, Feretzaki M, Xue C, Heitman J. Transcription factors Mat2 and Znf2 operate cellular circuits orchestrating opposite- and same-sex mating in *Cryptococcus neoformans*. PLoS genetics. 2010;6(5):e1000953. Epub 2010/05/21. doi: 10.1371/journal.pgen.1000953. PubMed PMID: 20485569; PubMed Central PMCID: PMCPmc2869318.

12. Nielsen K, Cox GM, Wang P, Toffaletti DL, Perfect JR, Heitman J. Sexual cycle of Cryptococcus neoformans var. grubii and virulence of congenic a and alpha isolates. Infect Immun. 2003;71(9):4831-41. Epub 2003/08/23. PubMed PMID: 12933823; PubMed Central PMCID: PMCPmc187335.

13. Lin X, Hull CM, Heitman J. Sexual reproduction between partners of the same mating type in *Cryptococcus neoformans*. Nature. 2005;434(7036):1017-21. Epub 2005/04/23. doi: 10.1038/nature03448. PubMed PMID: 15846346.

14. Zhai B, Zhu P, Foyle D, Upadhyay S, Idnurm A, Lin X. Congenic strains of the filamentous form of *Cryptococcus neoformans* for studies of fungal morphogenesis and virulence. Infect Immun. 2013;81(7):2626-37. Epub 2013/05/15. doi: 10.1128/iai.00259-13. PubMed PMID: 23670559; PubMed Central PMCID: PMCPmc3697605.

15. C. Rocha (National Research Council Canada)
