# Supplementary material for: Glucosamine stimulates pheromone-independent dimorphic transition in Cryptococcus neoformans by promoting Crz1 nuclear translocation
Source: PLoS Genet. 2017 Sep 12;13(9):e1006982. doi: 10.1371/journal.pgen.1006982 (PMC5595294; doi:10.1371/journal.pgen.1006982)
Supplement: S3 Table — (DOCX) [file pgen.1006982.s011.docx]

Supplemental Table 3. Oligonucleotides Used in This Study.

| Primers | Sequence (5’🡪 3’) |
| --- | --- |
| linlab3397/XX | TAATGGCCGGCCATGGCAGATCCAGCCTCACC |
| linlab3398/XX | TAATGCGATCGCATCCTCTTCACTCGTTTCACTCTTC |
| linlab3526/XX | TGATCTGGCCGGCCATGGCAGATCCAGCCTCACC |
| linlab3527/XX | CGGCGCTTAATTAATTATTAATCCTCTTCACTCGTTTCACTCTTC |
| linlab3653/XX | TAATGCGGCCGCGGTTCGTTAGTCGGGTCAACTG |
| linlab3654/XX | TAATGGCCGGCCCCTCTTCACTCGTTTCACTCTTCTTC |
| linlab3834/XX | CCAGCGTAGCGACAGAAGTG |
| linlab3835/XX | TCGCAAACCCTCATGCAGAAG |
| linlab3915/XX | GAAGGTAGTGGCAGACGGCTAAG |
| linlab3916/XX | ACCCTTGGTCACCTTCAGCTTG |
| LinLab327/XL | CTCTGGTTGGCACGGTG |
| LinLab328/XL | CGTCGGTCAATCTTCTCG |
| LinLab331/XL | GGAGGACCTTGAGAGTGAAG |
| LinLab332/XL | GAACGTCTTCTCTTTGTGGTG |
| LinLab333/XL | GCTCAACGTCACATCAAGC |
| LinLab334/XL | CTTGTGGCTCGTCAAAATG |
| Linlab577/XL | GCCTTCACTGCCATCTTC |
| Linlab578/XL | TTAGGCGATGACGCATAG |
| linlab1298/Wang | TGATCTGGCCGGCCAATCAAAATGGTGAGCAAGGGCGAGGA |
| linlab1299/Wang | TCACTCACGTCAAGAACGTCCTTGTACAGCTCGTCCATGCCG |
| linlab1341/Wang | CTCCACTCTCGTGCTCCTGAA |
| linlab1342/Wang | AGTTCGCTTGCCTTTTCCTTT |
